# Synthesis, In Vitro and In Silico Anticancer Activity of New 4-Methylbenzamide Derivatives Containing 2,6-Substituted Purines as Potential Protein Kinases Inhibitors

**DOI:** 10.3390/ijms222312738

**Published:** 2021-11-25

**Authors:** Elena Kalinichenko, Aliaksandr Faryna, Tatyana Bozhok, Alesya Panibrat

**Affiliations:** Institute of Bioorganic Chemistry, National Academy of Sciences of Belarus, 5/2 Academician V.F. Kuprevich Street, BY-220141 Minsk, Belarus; farina@iboch.by (A.F.); tbozhok@iboch.by (T.B.); panibrat@iboch.by (A.P.)

**Keywords:** purine derivatives, anticancer activity, protein kinase inhibitors, cellular apoptosis, molecular docking

## Abstract

A novel class of potential protein kinase inhibitors **7**–**16** was synthesized in high yields using various substituted purines. The most promising compounds, **7** and **10,** exhibited inhibitory activity against seven cancer cell lines. The IC_50_ values for compounds **7** and **10** were 2.27 and 2.53 μM for K562 cells, 1.42 and 1.52 μM for HL-60 cells, and 4.56 and 24.77 μM for OKP-GS cells, respectively. In addition, compounds **7** and **10** dose-dependently induced the apoptosis and cell cycle arrest at G2/M phase, preventing the cell division of OKP-GS cells. Compounds **7**, **9,** and **10** showed 36–45% inhibitory activity against PDGFRα and PDGFRβ at the concentration of 1 μM. Molecular modeling experiments showed that obtained compounds could bind to PDGFRα as either type 1 (compound **7**, ATP-competitive) or type 2 (compound **10**, allosteric) inhibitors, depending on the substituent in the amide part of the molecule.

## 1. Introduction

The discovery of imatinib, a selective inhibitor of Bcr–Abl protein kinase, in the late 1990s, was an important step not only in revealing the relationship between the Philadelphia chromosome and the onset of chronic myeloid leukemia, but also in the development of targeted cancer therapy in general [1,2]. The success of imatinib showed how effective a strategy of attacking a specific biological target in cancer cells can be and, no less important, drew attention to protein kinases as a key element of the cell signaling system, to which the action of targeted drugs can be directed [3,4]. More than 20 years have passed since the registration of imatinib, and every year the number of protein kinase inhibitors used for the treatment of cancer is only increasing [5,6]. In addition, the number of protein kinase targets for approved inhibitors is constantly expanding as well [7,8,9,10,11]. Recent progress also shows that protein kinase inhibitors can be used to treat non-oncological diseases [12,13]. It is important to emphasize that the known kinase targets constitute only a small part of the human kinome.

It should be noted, that, in most cases, the use of small-molecule kinase inhibitors (SMKIs) still does not provide as favorable a prognosis as it does in the case of imatinib and other Abl inhibitors. However, their relatively high efficacy, combined with good tolerance, makes them, in most cases, the first line of drug treatment [14]. The common causes of the primary or subsequent resistance to SMKIs are the mutations of their protein targets due to the genomic instability of cancer cells or the specificity of a particular patient [15]. Mutations change the structure of the binding site of a kinase, reducing the affinity of the inhibitor. Some of the most problematic mutations (for example, T315I for Abl kinase) make the binding completely impossible [16,17,18,19,20]. It should be noted that all kinases use the same substrate (ATP) and in general share many similarities in their structure. In most cases, an inhibitor targets several kinase enzymes at once. Such multi-targeting could be a positive moment for the cases when cancer cells over-express several protein kinases. However, blocking non-oncogenic proteins could lead to side effects [21]. Modulating drug selectivity is a major challenge in SMKI development.

X-ray structural data make it possible to distinguish a number of features of SMKI binding in a protein active site [22]. Most of the kinase inhibitors discovered to date are ATP-competitive and are classified as type 1 inhibitors. This type of inhibitor binds to the active conformation of a kinase. Type 1 inhibitors usually consist of a heterocyclic ring system that occupies the purine binding site, where it serves as a framework for its substituents to occupy adjacent hydrophobic regions I and II [23]. At the same time, a significant portion of inhibitors, including imatinib, bind to a kinase that is in a biologically inactive conformation (type 2 inhibitors), in which the flexible kinase activation loop opens an additional, allosteric pocket. This pocket can be used by an inhibitor to form additional interactions. Common pharmacophore features of type 2 inhibitors are: the presence of a specific heterocyclic base creating interactions in the ATP pocket, and the amide bond, usually with a 3-trifluoromethylaniline substituent, which provides strong interactions in the allosteric site. The linker that connects these two fragments is, as a rule, represented by a benzene ring with substituents at different positions [24].

In our previous work, we suggested to use a flexible 4-methylbenzamide linker to obtain new chemical compounds capable of inhibiting protein kinases. A number of the obtained derivatives of 4-methylbenzamide showed high biological activity in vitro and in silico [25,26].

In this study, the design, synthesis and study of the in vitro biological activity of new 4-methylbenazmide derivatives were carried out. The key idea of the design of new compounds is to preserve the N-(3-trifluoromethyl-phenyl)-4-methylbenzamide backbone, and to use various purine derivatives as substituents of the methyl group of 4-methylbenzamide. The N-9 atom of a purine was used for this connection. On the one hand, flexible linker and purines as a heterocyclic base provide the pharmacophore similarity of the target compounds with known type 2 SMKIs. On the other hand, purine derivatives can imitate the natural ATP substrate, being potential type 1 kinase inhibitors.

In this investigation, an attempt is made to trace the evolutionary pathway of purine-containing analogs by a systematic search for structural and electronic similarities, using an in silico method for screening the obtained compounds with a number of different protein kinases and to assess the contribution of modified purine bases and imidazole groups to binding to the active site of various protein kinases.

## 2. Results and Discussion

### 2.1. Molecular Design

**Figure 1:**
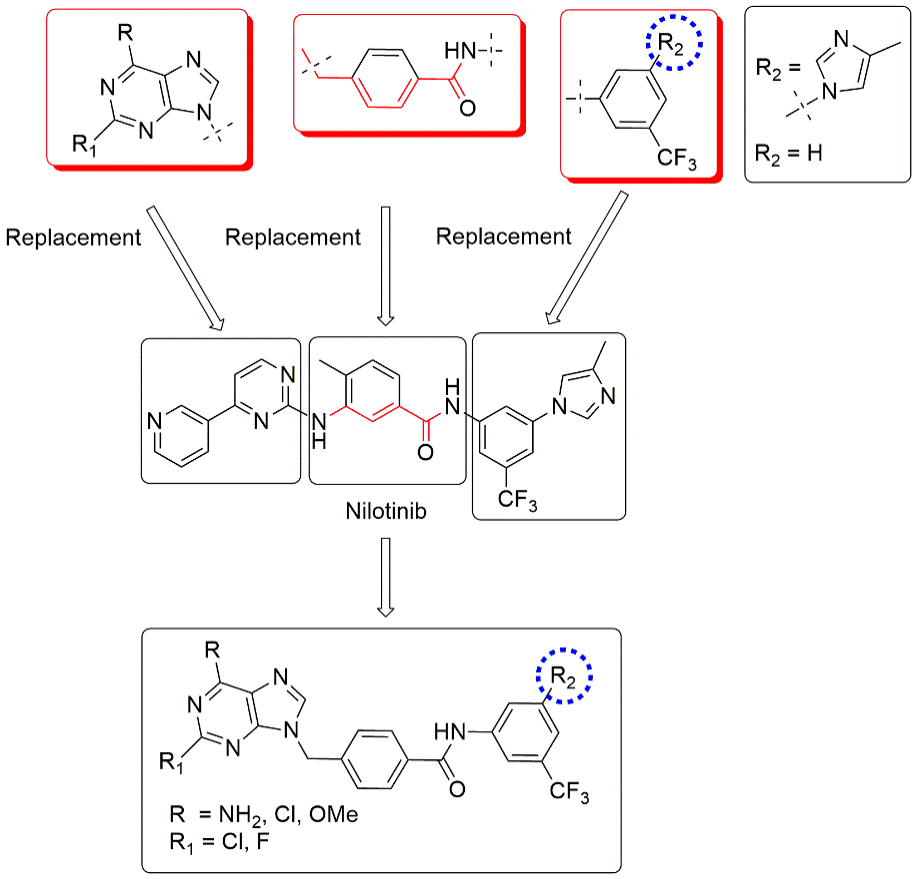
Design strategy and structures of title compounds.

Based on the structural analysis of type 2 SMKI, we began the development of new potential Bcr–Abl inhibitors using the combination of bioisosteric substitution and conformational restriction. Nilotinib, a second-generation Bcr–Abl inhibitor, was used as base structure. Structural modifications were focused on the replacement of the phenylaminopyrimidine, which forms hydrogen bonds with the amino acids Met-318 and Thr-315 in the adenine pocket, with 2,6-substituted purines (Figure 1). As a linker between the adenine pocket and the DFG-out pocket, we used 4-(aminomethyl)benzamide, the derivatives of which showed a significant in vitro and in silico anti-kinase activity in previous studies [24].

3-(4-Methyl-1H-imidazol-1-yl)-5-(trifluoromethyl)aniline was chosen as the terminal fragment considering the additional interactions that are formed due to the presence of a trifluoromethyl substituent in the phenyl ring. This allow nilotinib to penetrate deeper into the allosteric pocket, enhancing hydrophobic interactions around the phenyl group and the imidazole fragment, which is in close contact with the Phe-359 residue in the C-lobe [27]. To clarify the importance of these interactions, the imidazole fragment was replaced in some structures by a hydrogen atom (Figure 1).

Based on these design principles, ten new analogs have been developed, synthesized, and studied as potential inhibitors of cell proliferation and protein kinase activity.

### 2.2. Chemistry

**Scheme 1:**
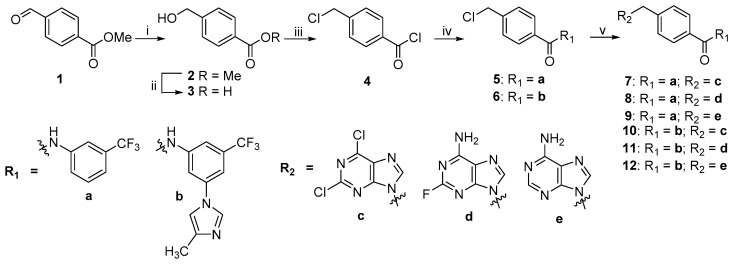
Synthetic route of derivatives **7**–**12**. Reagents and conditions: (**i**) NaBH_4_, MeOH, rt, 2 h; (**ii**) KOH, MeOH/H_2_O, 60 °C, 2 h; (**iii**) SOCl_2_, CHCl_3_, reflux, 5 h; (**iv**) amine R_1_H (**a**/**b**), DMF, CHCl_3_, rt; (**v**) purine base R_2_H (**c**/**d**/**e)**, K_2_CO_3_, DMF, 80–90 °C.

The synthesis of starting compounds **2**–**4** was carried out according to previously reported methods [28,29]. Compounds **5** and **6** were obtained by reacting chlorobenzoate **4** with 3-(trifluoromethyl) aniline or 3-(4-methyl-1H-imidazol-1-yl)-5-(trifluoromethyl) aniline, followed by isolation by column chromatography as described in [25]. Compounds **7**–**12** were synthesized by the condensation of the corresponding chlorine derivative **5** and **6** with the potassium salt of the purine analogue **c**–**e**, obtained by treating the corresponding base with potassium carbonate in dimethylformamide at reflux, in high yields (Scheme 1).

**Scheme 2:**
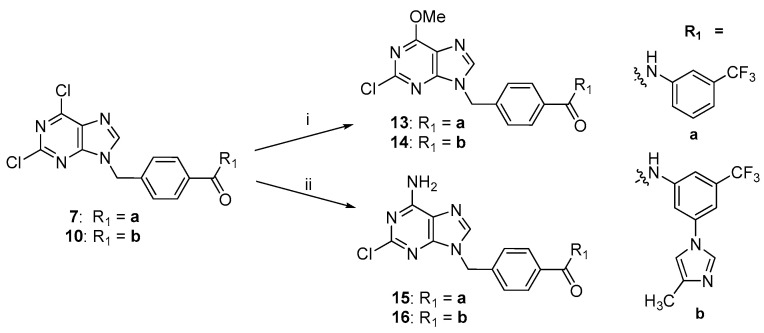
Synthetic route of derivatives 13–16. Reagents and conditions: (**i**) MeOH, K_2_CO_3_, 50 °C, 30 min; (**ii**) 25% aq. NH_4_OH, CH_3_CN, 50 °C, 1 h.

The treatment of 2,6-dichloropurine derivative **7** or **10** with potassium carbonate in dry methanol led to the formation of purine analogs **13** and **14** with a yield of 69% and 45%, respectively (Scheme 2). Analogs **15** and **16** were obtained from the corresponding compounds **7** or **10** by treatment with 25% aq. NH_4_OH in high yields.

The structures of synthetic intermediates and products were established by ^1^H, ^13^C, and ^19^F NMR spectroscopy and high-resolution mass spectrometry (HRMS). In the ^1^H NMR spectra of compounds **12**–**16**, there are chemical shifts in the range 5.45–5.62 ppm, which are typical proton signals of the methylene group connected to the N-9 nitrogen atom of the imidazole ring of purine. In comparison, for related compounds containing a CH_2_ group attached not to a purine, but to a secondary amine, the proton signal is shifted to a strong field and is located in the range of 4.54–4.69 ppm [25]. In the case of analogs containing a CH_2_ group at the piperazine ring, an even stronger shift of proton signals in a strong field up to 3.62 ppm is observed [25]. In the ^19^F NMR spectra of compounds **8** and **11**, an additional signal appears at −52.1 ppm, related to the fluorine atom of the purine moiety besides the CF_3_ signal at −61.3 ppm. Graphical NMR spectra of target compounds can be found in the Supplementary Materials.

### 2.3. Biological Studies

#### 2.3.1. Anti-Proliferation Activity

To determine the selectivity of the synthesized compounds **7**–**16** toward various cancer cells and normal human cells, the anti-proliferative activity against K562 (chronic myelogenous leukemia), HL-60 (promyelocytic leukemia), MCF-7 (breast adenocarcinoma), HeLa (cervix carcinoma), HepG2 (human liver cancer), A549 (lung carcinoma), OKP-GS (renal cell carcinoma), and normal cells RPMI 1788 (B lymphocyte, the human cell line) was investigated.

The data obtained for compounds **7**–**16** are provided in Table 1. Known protein kinase inhibitors were used as reference compounds: imatinib, sorafenib and nilotinib. For imatinib, the IC_50_ value for the chronic myeloid leukemia cell line (K562) turned out to be less than 1 μM, which is consistent with the data hereof [30].

The compounds **7** and **10**, containing two chlorine atoms at the C-2 and C-6 positions of the purine heterocycle, demonstrated high activity against all studied cell lines, comparable to the data for sorafenib. The highest activity was observed against the leukemic cell line K562 with IC_50_ values of 2.27 and 2.53 µM, and IC_50_ values for HL-60 equal to 1.42 and 1.52 µM, respectively.

The compound **7** showed high activity against the OKP-GS renal carcinoma cell line with an IC_50_ value of 4.56 µM. Additionally, a significant inhibitory ability against OKP-GS was found in compounds **13** and **14** containing a chlorine atom and a methoxy group at the C-2 and C-6 positions of the purine fragment, respectively. It should be noted that the absence of a 4-methyl-imidazole fragment in structures **7** and **13** had no noticeable effect on their antiproliferative activity against the studied cell lines as compared with analogs **10** and **14**.

According to the data in Table 1, the synthesized compounds were toxic to the normal cell line RPMI 1788, comparable to the results for reference compounds that inhibited the growth of normal human dermal fibroblasts (NHDF) in experiments in vitro [31,32].

#### 2.3.2. Kinase Inhibitory Assays

The Bcr–Abl1 inhibitory activity assay was performed using Abl1 Kinase Enzyme System (Promega, Madison, WI, USA) and ADP-Glo™ Kinase assay kit (Promega, Madison, WI, USA). Compound **10**, containing 2,6-dichloropurine and 3-(4-methyl-1H-imidazol-1-yl)-5-(trifluoromethyl) aniline fragment, showed inhibitory activity against Bcr–Abl1 kinase. The rest of the compounds showed moderate inhibitory activity in the studied concentration range (Table 2).

Considering the presence of a purine ring in the compounds under study, type 1 binding to protein kinases could be expected. We investigated the kinase selectivity of targeted compounds **7**–**16** against eight receptor tyrosine kinases including EGFR, HER2 and HER4 (epidermal growth factor receptors), IGF1R (insulin-like growth factor 1 receptor), InsR (insulin receptor), KDR (vascular endothelial growth factor receptor 2—VEGFR-2), and PDGFRα and PDGFRβ (platelet-derived growth factor receptors). The assay was performed using the Kinase Selectivity Profiling System TK-1 (Promega, Madison, WI, USA) and ADP-Glo™ Kinase assay kit (Promega, Madison, WI, USA). Kinase activity was measured by quantifying the amount of ATP remaining in solution following a kinase reaction. Enzymatic activity inhibition percentage caused by studied compounds was evaluated at the single concentration of 1 μM. Lapatinib and sorafenib, which are type 2 inhibitors of TK 1, were taken as comparison compounds. The obtained results are given in Table 3.

Compounds **7, 9** and **10** showed good inhibition of PDGFRα and PDGFRβ in the range of 36 to 45%. To a smaller extent, 22–26% inhibition of HER2 kinase by analogs **7** and **8** was observed compared with the control substances (Table 3).

### 2.4. Cell Apoptosis Assay of Compounds ***7*** and ***10***

**Figure 2:**
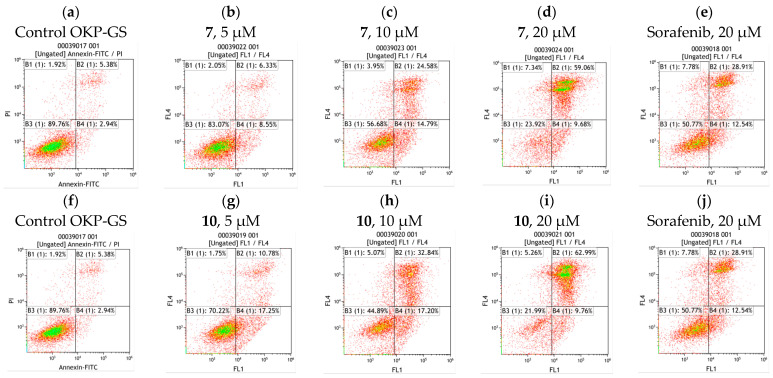
Effects of compounds **7** and **10** on apoptosis in OKP-GS cells compared with the positive control sorafenib. Representative flow cytometry dot plots showing the % of specific cell populations (viable, early apoptosis and late apoptosis) in OKP-GS cells (**a**,**f**); treated with compound 7 at a concentration of 5.0 (**b**), 10.0 (**c**), 20.0 μM (**d**) and sorafenib 20.0 μM (**e**) for 72 h; treated with compound **10** at a concentration of 5.0 (**g**), 10.0 (**h**), 20.0 μM (**i**) and sorafenib 20.0 μM (**j**) for 72 h.

Apoptosis is a versatile and extremely effective pathway for cell death. This phenomenon can be caused by internal signals, such as genotoxic stress, or external signals, such as the binding of ligands to death receptors on the cell surface [33]. The induction of apoptosis was studied by measuring the translocation of phosphatidylserine from the cytoplasmic to the extracellular side of the plasma membrane. OKP-GS cells were treated with compound **7** or **10** at 5, 10 and 20 μM for 72 h, after which annexin-V/propidium iodide (PI) binding was measured by flow cytometry (Figure 2).

**Figure 3:**
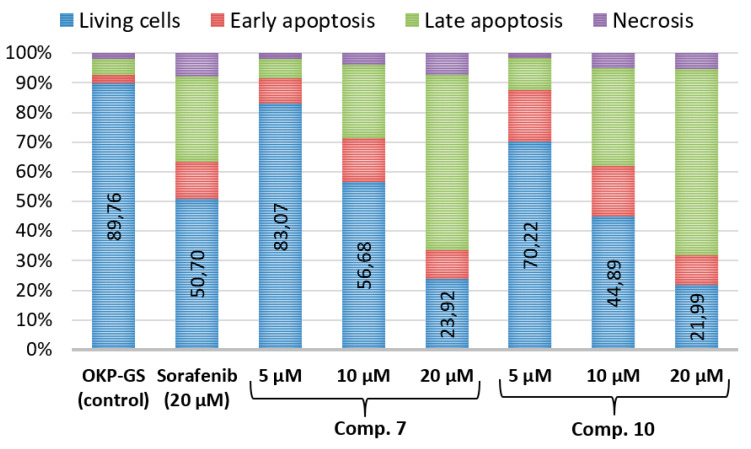
Apoptosis data analysis of OKP-GS cells treated with compounds **7** and **10**.

The data obtained show that analogs **7** and **10** at a concentration of 10 μM have a significant effect on the induction of apoptosis, comparable to sorafnib at a concentration of 20 μM. At a concentration of 20 μM, compounds **7** or **10** exhibited a more than two-fold higher level of apoptosis compared with sorafnib. Compounds **7** and **10** were found to promote apoptosis in a dose-dependent manner (Figure 3). The number of necrotic cells increased almost linearly and did not exceed 7.3%, which suggests that after 72 h cell death is mainly due to apoptosis, not necrosis.

### 2.5. In Vitro Cell Cycle Effects of Compounds ***7*** and ***10***

**Figure 4:**
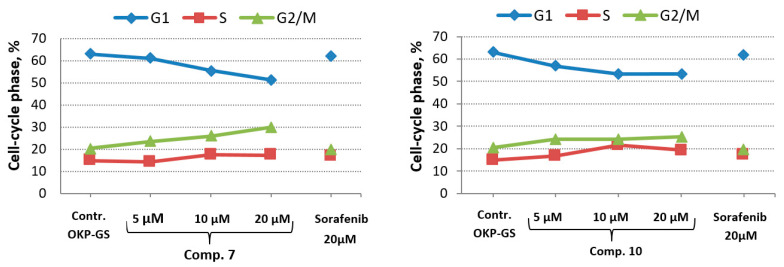
Cell cycle inhibition of OKP-GS cells treated with different concentrations of compounds **7, 10** and 20 µM of sorafenib as a reference compound.

Cell cycle experiments were performed with OKP-GS cells treated with 5.0 μM, 10 μM and 20 μM compounds **7**, **10** and 20 μM sorafenib, respectively (Table 4, Figure 4). To assess changes in the distribution of cells by phases of the cell cycle, we used the method of staining fixed samples with propidium iodide (Calbiochem, USA). Pre-fixation of cells was carried out with 70% ethanol for 72 h. To exclude the binding of propidium iodide to RNA, the fixed samples were treated with RNAse A at a final concentration of 50 μg/mL (R4875, Sigma, St. Louis, MI, USA) for 15 min at 37 °C [34,35].

Cell cycle analysis showed a significant increase in the number of OKP-GS cells in the G2/M phase from 20.43 (DMSO) to 23.53, 26.02 and 29.94% after treatment with compound **7** at 72 h at the concentrations of 5, 10 and 20 μM, respectively. In the G1 phase, the number of OKP-GS cells decreased linearly and was 51.2% at a maximum dose of 20 μM (Figure 4, Table 4). For all studied substances the number of cells in the S phase was slightly increased in comparison with the control when treated at a concentration of 10 μM and 20 μM. The obtained result for compounds **7** and **10** showed that cells were able to progress from the G1 phase to the S phase, being then blocked in the G2/M phase. That caused a decrease in the number of cells in the G1 phase and an increase in the G2/M phase with an almost constant number of cells in the S-phase. Based on the results, we came to the conclusion that compound **7** dose-dependently blocks cells in the G2/M phase.

Compound **10** suppressed the development of the cell cycle in a similar way, although in a less pronounced form. At the same time, the effect of compounds **7** and **10** on cell cycle progression, leading to a decrease in OKP-GS cell proliferation, was more significant than that of sorafenib at 20 μM. The cell cycle analysis of OKP-GS cells is shown in Figure 4. The results for compounds **7** and **10** are summarized in Table 4.

### 2.6. Docking and Molecular Dynamics

The anticancer activity of the obtained compounds was evaluated in silico using Autodock Vina [36] as the most popular open source docking software. As receptors for docking, we used 33 experimental structures of cancer-related protein targets, most of which were protein kinases (Abl, Src, Aurora). Two receptors were poly (ADP-ribose) polymerases (PARP), since this class of proteins is a promising target in the treatment of cancer. Three-dimensional models of these proteins in complexes with known inhibitors were taken from the Protein Data Bank (PDB) [37].

The potential inhibitory activity of the synthesized derivatives of 4-methylbenazmide was evaluated based on the comparison of their docking scores with the docking scores of known inhibitors from PDB complexes. Structures of native inhibitors were docked twice. Initially, the original three-dimensional structure of the original ligand from the PDB file was used. In the second experiment, all original ligand structures were preliminarily minimized using a GAFF force field, which aims to simulate constructing them from scratch. For a number of receptors, docking scores for native ligands were taken from our previous works [25,26]. The correctness of the reproduction of the experimental docking poses for original ligands was assessed visually by the comparison with the ligands contained in the PDB complexes.

The values of the obtained docking scores for original ligands and studied structures are provided in Table 5. For the known ligands, the difference in binding energies for minimized and non-minimized structures was found to be negligible. In addition, for correctly reproduced poses, the docking scores were significantly better than for poses that did not correspond to the real binding model.

It should be noted that for the complexes of type 2 inhibitors, in which the ligand occupies the ATP pocket, allosteric pocket, and linker cavity, Autodock Vina made it possible to correctly reproduce the position of the inhibitor in all cases. Good results were also obtained for PARP receptors. For complexes of known inhibitors of type 1 or mixed type, which do not occupy the allosteric pocket, the docking error was quite high. The arrangement of complex aromatic systems often coincided with the experiment (PDB: 3ZBF, 4C2W, 5EW9, 6NEC), but the flexible parts of the structures were not docked properly. For example, for the complex of Aurora kinase with ATP (PDB: 4C2W), AutoDock Vina correctly identified the location of the purine and sugar fragments, but triphosphate residue did not match the original ligand. The strongest discrepancies between the predicted docking pose and experiment were observed for complexes 2BFY, 2VRX, 4B8M, and 4G5P.

The best docking scores were obtained for complexes of **7**–**9**, **13** and **15**, which do not contain a 4-methyl-imidazole fragment in their structure, with protein kinases of the epidermal growth factor family, HER2, ErbB4, VEGFR1, and EGFR T790M, as well as with kinase TRKb of the neurotrophin receptor group (Trk), which suggests the presence of inhibitory activity against these enzymes (Table 5).

For protein kinases Aurora A and Aurora B, the docking energies were from −8.7 to −10.4 kcal/mol, which corresponded to or exceeded the original ligand. 

To refine the binding energy, a number of molecular dynamics experiments were carried out to calculate the binding energy and its components for complexes of the studied structures and some known inhibitors as reference. The binding energy calculations were performed using the MM-PBSA method [38] on the frames of the trajectory obtained after 2 ns molecular dynamics simulation, which was carried out using GROMACS [39]. The obtained binding energies are provided in Table 6.

The studied compounds demonstrated, in general, lower binding energies, in comparison with the results of known inhibitors obtained in this work and in previous studies [25]. Complexes of compounds **10**–**12** with Abl kinase (PDB: 3CS9) showed high values of VDW component in binding energy, but the energy of electrostatic interactions turned out to be low. High values of electrostatic interactions were found for complexes of **8**, **9** and **15** with BRAF kinase (PDB: 5HI2).

### 2.7. Molecular Modeling Study

To better understand the results of molecular docking and data on kinase inhibition for compounds **7**–**16**, several complexes of target compounds with various proteins were visualized using the Chimera software [41].

The synthesized compounds can be considered as hybrid molecules containing various substituted purines as heterocyclic bases attached at the N-9 position through a flexible linker to the N-(3-trifluoromethyl-phenyl)-4-methylbenzamide fragment (**7**–**9**, **13**, **15**) or to N-3-(4-methyl-imidazol-1-yl)-5-(trifluoromethyl)phenyl)-4-methylbenzamide (**10**–**12**, **14**, **16**). On the one hand, this design makes it possible to ensure the pharmacophore similarity of the target compounds with ATP-competitive inhibitors, and on the other hand, to imitate type 2 SMKIs. Complexes of studied structures with proteins PDGFRα (PDB: 6JOL) [42], Abl (PDB: 3CS9) [43], BRAF (PDB: 5HI2) [44], VEGFR1 (PDB: 3HNG) [45], and Aurora B (PDB: 2BFY) [46] were chosen for visualization.

Most of the known type 1 kinase inhibitors form one to three hydrogen bonds with amino acids located in the hinge region of a kinase, thereby mimicking the hydrogen bonds that are usually formed by the adenine ring of ATP [47,48]. They generally do not use a ribose-binding pocket or triphosphate-binding pocket.

**Figure 5:**
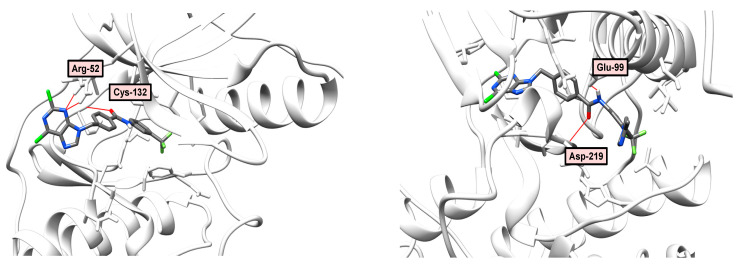

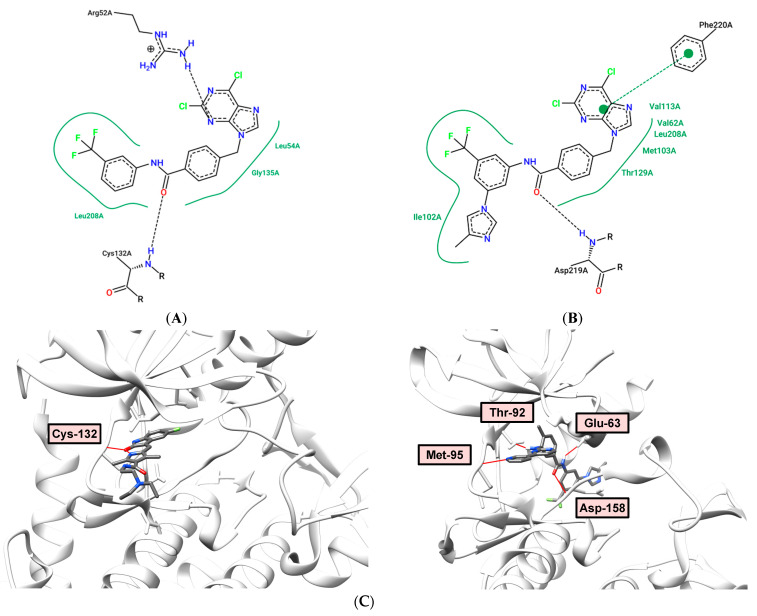
(**A**) Binding model (top) and 2D interaction map (bottom) of **7** to PDGFRα (PDB: 6JOL) kinase. H-bond—Arg-52, Cys-132; (**B**) Binding model (top) and 2D interaction map (bottom) of **10** to PDGFRα (PDB: 6JOL) kinase. H-bond—Glu-99, Asp-219, Phe-220; type 2 binding; (**C**) Comparison of binding modes of type 1 (lapatinib PDB: 6JOL, H-bond Cys-132, left) and type 2 (nilotinib, PDB: 3CS9, H-bond—Glu-63, Asp-158, Thr-92, Met-95, right).

As shown in Figure 5A, compound **7** occupies the PDGFRα kinase ATP binding site (PDB: 6JOL). In this binding model, Arg-52 forms two hydrogen bonds with the N-3 purine atom (distances: 2.47 and 2.08 Å). In addition, the NH_2_ hydrogen of the Cys-132 group serves as an H-bond donor for the amide group of 4-metylbenzamide (distance: 1.71 Å).

In the case of dichloro compound **10**, the structure behaves as a type 2 inhibitor, with a purine fragment being located in the adenine pocket, and the allosteric region is occupied by the trifluoromethyl and imidazole substituents of the phenyl ring. The amide group forms two hydrogen bonds with Glu-99 (distance: 1.97 Å) and Asp-219 (distance: 2.10 Å), as shown in Figure 5B. Compound **10** is characterized by a higher calculated binding energy (-133.473 kJ/mol) to receptor tyrosine kinase PDGFRα (PDB: 6JOL) than compound **7** (−109.837 kJ/mol), as shown in Table 6. Figure 5C shows differences in type 1 (lapatinib) and type 2 (nilotinib) inhibitors binding. Lapatinib binds to the ATP-binding site, not touching allosteric and linker pockets. Nilotinib occupies all three binding site regions: pyrimidine imitates ATP, 3-(4-methyl-1H-imidazol-1-yl)-5-(trifluoromethyl)aniline rests in the allosteric pocket, central benzamide ring implements proper orientation for these two fragments also forming hydrogen bonds with aspartic and glutamic residues. In fact, these two hydrogen bonds can be seen in the majority of type 2 inhibitor complexes.

**Figure 6:**
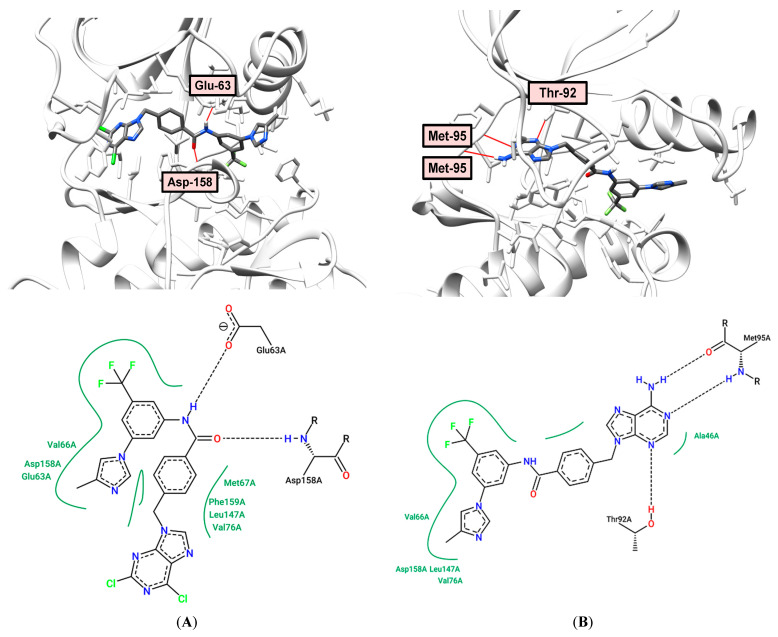
(**A**) Binding model (top) and 2D interaction map (bottom) of **10** to Abl (PDB: 3CS9) kinase. H-bond—Glu-63A, Asp-158A; (**B**) Binding model (top) and 2D interaction map (bottom) of **12** to Abl (PDB: 3CS9) kinase. H-bond—Thr-92A, Met-95A.

Comparing the binding models of dichloro derivative **10** and purine analogue **12** to Abl-kinase (PDB: 3CS9), it should be noted that their positions are typical for type 2 inhibitors. The presence of the adenine ring in compound **12** leads to the formation of three hydrogen bonds in the adenine pocket. The amino group of purine (distance: 2.24 Å) and the N-1 purine atom (distance: 2.27 Å) interact with the Met-95 residue, the N-3 atom of purine forms hydrogen bond with Thr-92 (distance: 2.13 Å), as shown in Figure 6B. At the same time, the presence of two chlorine atoms in analogue **10** does not allow the formation of hydrogen bonds in the hinge (Hinge) region, but the amide group forms two hydrogen bonds with residues Glu-63 (distance: 2.26 Å) and Asp-258 (distance: 1.86 Å), as shown in Figure 6A. However, compound **10** has a higher calculated binding energy (−144.216 kJ/mol) to Abl tyrosine kinase (PDB: 3CS9) than **12** (−137.285 kJ/mol), and a high antiproliferative activity against a number of cell lines (Table 1 and Table 6).

**Figure 7:**
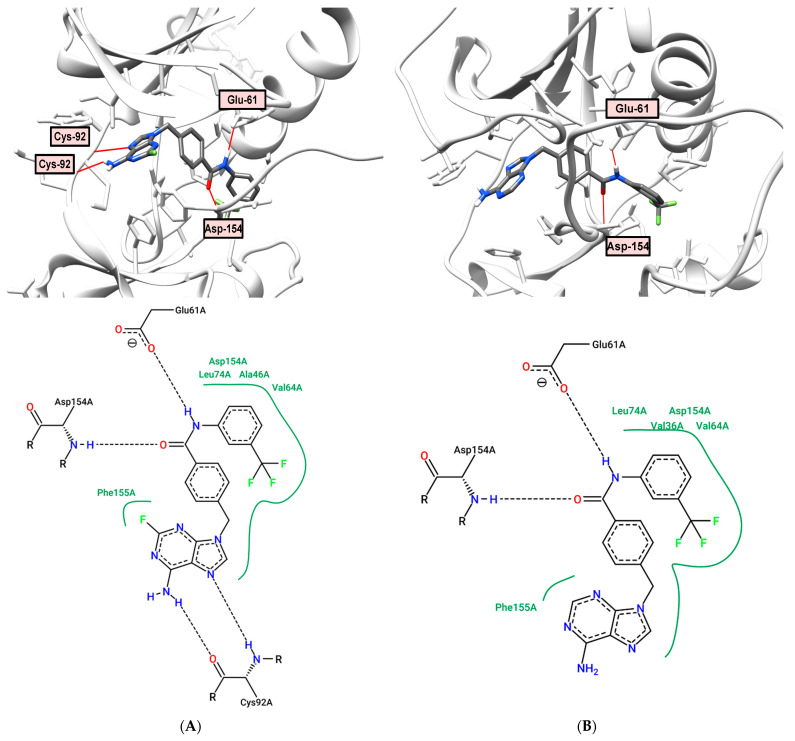
(**A**) Binding model (top) and 2D interaction map (bottom) of **8** to BRAF (PDB: 5HI2) kinase. H-bond—Glu-61, Cys-92, Asp-154; (**B**) Binding model (top) and 2D interaction map (bottom) of **9** to BRAF (PDB: 5HI2) kinase. H-bond—Asp-154, Glu-61.

The position of the 2-fluoroadenine derivative **8** in the active site of the BRAF (PDB: 5HI2) kinase is more consistent with type 1 inhibitors (Figure 7A). Binding of compound **8** revealed four hydrogen bonds: the proton of the N-6 amino group as an H-donor (distance 1.91 Å), and the N-7 adenine atom as an acceptor (distance 2.28 Å) binds to the Cys-92 residue, while the amide group forms third and fourth H-bonds with Glu-61 (distance: 2.29 Å) and Asp-154 (distance: 1.76 Å). Despite the formation of four hydrogen bonds, compound **8** has a low calculated binding energy (−95.507 kJ/mol) with respect to BRAF kinase and did not show significant activity against the studied cell cultures and cancer-related kinases (Table 1, Table 2 and Table 3).

The BRAF-binding domain in Figure 7B demonstrates that the amide group of adenine derivative **9** participates in two H-bonds with side chains residues Glu-61 and Asp-154 (distance: 1.90 and 2.14 Å, respectively). The adenine fragment does not participate in the formation of hydrogen bonds, unlike 2-fluoroanalog **8**. Purine analog **9** showed good inhibitory activity against PDGFRα PDGFRβ receptor kinases, 45% and 36%, respectively, at a concentration of 1 μM (Table 3).

**Figure 8:**
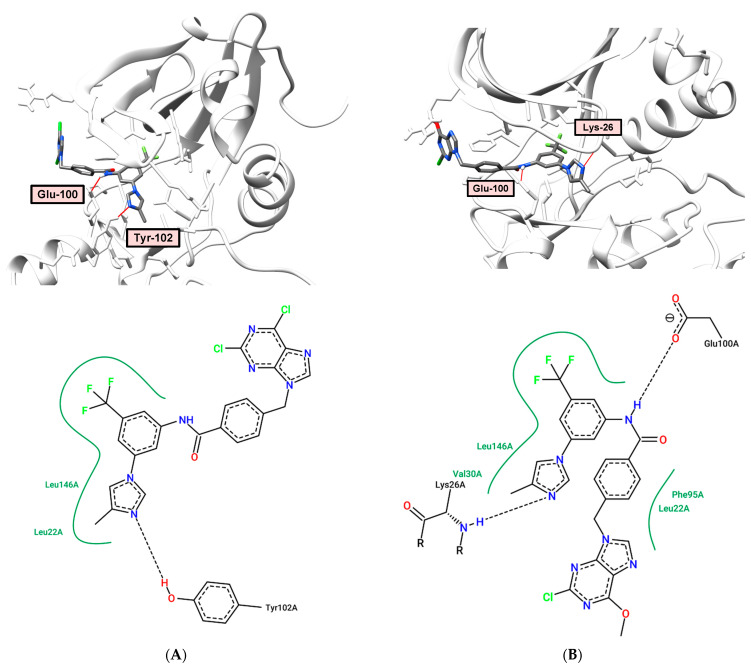
(**A**) Binding model (top) and 2D interaction map (bottom) of **10** to Aurora-B (PDB: 2BFY) kinase. H-bond—Glu-100, Tyr-102; (**B**) Binding model (top) and 2D interaction map (bottom) of **14** to Aurora-B (PDB: 2BFY). H-bond—Glu-100, Lys-26.

The binding model of compound **10** to Aurora B kinase (PDB: 2BFY) is mediated by two hydrogen bonds, as shown in Figure 8A. H-bond interactions can be seen between the hydrogen of the NH amide group and the main chain residue Glu-100 (distance: 2.49 Å). Another H-bond is formed by Tyr-102, which acts as a hydrogen bond acceptor, with the N-3 atom of the 4-methylimidazole ring (distance: 2.30 Å).

In the case of 6-OMe-2-Cl-purine derivative **14**, a similar arrangement of the ligand in the adenine pocket of the Aurora-B kinase with the formation of two hydrogen bonds is observed (Figure 8B). As in the case of compound **10**, one H-bond is a donor–acceptor interaction between the hydrogen of the amide NH group and Glu-100 (distance: 1.86 Å). The N-3 atom of the 4-methylimidazole ring forms a hydrogen bond with Lys-26 (distance: 2.22 Å).

Obtained binding models, analysis of hydrogen bonds and binding energy calculations suggest that compounds **10** and **14** could bind to the active site of Aurora-B as type 1 inhibitors, despite the presence of a bulky N-(3-(4-methyl-1H-imidazol-1-yl)-5-(trifluoromethyl)phenyl) fragment.

## 3. Materials and Methods

### 3.1. Chemistry

Organic solvents were purified and dried by standard methods before usage in the synthesis. TLC analysis was carried out using TLC Silica gel 60 F_254_ (aluminium-backed sheets, Merck). The following solvent systems were used: CHCl_3_: MeOH, 7:1, *v*/*v* or EtOAc, isocratic. Preparative column chromatography was performed on silica gel Merck 60 (70–230 mesh). NMR spectra were registered on a Bruker Avance 500 MHz spectrometer at 500 MHz (^1^H), 125 MHz (^13^C) and 470 MHz (^19^F). Chemical shifts (*δ*) are given in ppm related to internal SiMe_4_ and coupling constants (*J*) in Hz. The signals were designated as follows: s, singlet; d, doublet; t, triplet; q, quartet; m, multiplet. High-resolution mass spectra (HRMS) were recorded on an Agilent 1290 Accurate-Mass 6500 Series Q-TOF using ESI (electrospray ionization). The purity of the synthesized compounds was determined by high-performance liquid chromatography (HPLC) (Waters, 996, Milford, MA, USA) with an EC 250/4,6 NUCLEODUR 100-5 C18ec column using KH_2_PO_4_ (0.02 M, pH = 6.8) with a formic acid/acetonitrile 45:55 mobile phase (1.0 mL/min). Melting points (m.p.) were determined on an electrically heated melting point apparatus and were uncorrected.

#### 3.1.1. Synthesis of 4-Methylbenzamide Intermediates **5** and **6**

The synthesis of starting compounds **2**–**4** was carried out according to the previously described methods [28,29].

A solution of amine 3-(trifluoromethyl)aniline or 3-(4-methyl-1H-imidazol-1-yl)-5-(trifluoromethyl)aniline (one equivalent) and N,N-dimethylformamide (DMF, 1.1 equivalents) in dry CHCl_3_ cooled to 0 °C was added dropwise to a solution of 4-(chloromethyl)benzoyl chloride (**4**) (one equivalent) in dry CHCl_3_. The reaction mixture was stirred at room temperature. The progress was monitored by TLC. Cold water was added to the reaction mixture. The organic layer was separated, and the water layer was extracted by CHCl_3_ three times. The combined organic layers were dried over Na_2_SO_4_, filtered and concentrated under vacuum. The product was purified by column chromatography on silica gel. The fractions containing the products **5** or **6** were collected and evaporated to dryness under vacuum. The residue was crystallized.

*4-(Chloromethyl)-N-(3-(trifluoromethyl)phenyl)benzamide* (**5**): yield 83%, white solid. ^1^H NMR (DMSO-d_6_): *δ* 10.60 (s, 1H), 8.27 (s, 1H), 8.07 (d, 1H, *J* = 8.3), 7.99–8.03 (m, 2H), 7.60–7.66 (m, 3H), 7.48 (d, 1H, *J* = 7.7), 4.88 (s, 2H). ^13^C NMR (DMSO-d_6_): *δ* 166.0, 141.9, 140.4, 134.7, 130.4, 130.0, 129.7, 129.3, 128.6, 125.7, 124.3, 120.5, 116.8, 45.8.

*4-(Chloromethyl)-N-(3-(4-methyl-1H-imidazol-1-yl)-5-(trifluoromethyl)phenyl)benzamide* **(6):** yield 80%; white solid. ^1^H NMR (DMSO-d_6_): *δ* 10.74 (s, 1H), 8.32 (s, 1H), 8.26 (d, 1H, *J* = 1.0), 8.17 (s, 1H), 8.03 (d, 2H, *J* = 8.3), 7.77 (s, 1H), 7.66 (d, 2H, *J* = 8.0,), 7.52 (s, 1H), 4.89 (s, 2H), 2.21 (s, 3H). ^13^C NMR (DMSO-d_6_): *δ* 166.1, 142.2, 141.7, 139.4, 138.4, 135.5, 134.4, 129.4, 128.6, 115.4, 114.7, 112.2, 45.8, 14.0.

#### 3.1.2. General Method for the Synthesis of Key Compounds **7**–**12**

To a solution of purine base (**c**/**d**/**e**) (1.1 equivalents) in dry DMF was added K_2_CO_3_. To this mixture, chloride **5** or **6** (one equivalent) in DMF was added under stirring. Then, the reaction mixture was stirred at 80–90 °C under nitrogen. The progress of the reaction was monitored by TLC. Then, the reaction mixture was brought to room temperature and diluted with CHCl_3_. The organic phase was washed with water, dried over Na_2_SO_4_ and evaporated to dryness. The product was purified by column chromatography on silica gel. The fractions containing the products **7**–**12** were collected and evaporated to dryness under vacuum. The residue was crystallized.

*4-((2,6-Dichloro-9H-purin-9-yl)methyl)-N-(3-(trifluoromethyl)phenyl)benzamide* (**7**): yield 56%, white solid, m.p. 249–251 °C. ^1^H NMR (DMSO-d_6_): *δ* 10.54 (s, 1H), 8.89 (s, 1H), 8.23 (s, 1H), 8.01 (d, 1H, *J* = 8.1), 7.96 (s, 1H), 7.94 (s, 1H), 7.59 (t, 1H, *J* = 8.0), 7.50–7.44 (m, 3H), 5.61 (s, 2H). ^13^C NMR (DMSO-d_6_): *δ* 165.4, 153.4, 151.1, 149.8, 148.4, 139.8, 139.3, 134.1, 130.5, 129.8, 129.3 (d, *J* = 31.3), 128.2, 127.5, 124.1 (d, *J* = 273.1), 119.9 (d, 2C), 116.2 (d, 2C), 46.8. ^19^F NMR (DMSO-d_6_): -61.29. HRMS (M + H)^+^ calcd. for C_20_H_12_Cl_2_F_3_N_5_O: 465.0371, found 466.0432.

*4-((6-Amino-2-fluoro-9H-purin-9-yl)methyl)-N-(3-(trifluoromethyl)phenyl)benzamide* (**8**): yield 78%, white solid, m.p. 309–311 °C. ^1^H NMR (DMSO-d_6_): *δ* 10.53 (s, 1H), 8.28 (s, 1H), 8.22 (s, 1H), 8.01 (d, 1H, *J* = 7.9), 7.94 (s, 1H), 7.92 (s, 1H), 7.84 (br.s, 2H), 7.59 (t, 1H, *J* = 7.9), 7.45–7.43 (d, 3H), 5.42 (s, 2H). ^13^C NMR (DMSO-d_6_): *δ* 165.5, 158.5 (d, *J* = 256.0), 157.8 (d, *J* = 30.0), 150.7 (d, *J* = 20.0), 141.2, 140.4, 139.8, 133.9, 129.8, 129.3 (d, *J* = 32.0), 128.1, 127.4, 124.1 (d, *J* = 273.3), 123.6, 119.9, 117.1, 116.2 (d, 2C), 45.9. ^19^F NMR (DMSO-d_6_): *δ* −61.27, −52.11; HRMS (M + H)^+^ calcd. for C_20_H_14_F_4_N_6_O: 430.1165, found 431.1231.

*4-((6-Amino-9H-purin-9-yl)methyl)-N-(3-(trifluoromethyl)phenyl)benzamide* (**9**): yield 93%, white solid, m.p. 293–295 °C. ^1^H NMR (DMSO-d_6_): *δ* 10.51 (s, 1H), 8.30 (s, 1H), 8.22 (s, 1H), 8.15 (s, 1H), 8.01 (d, 1H, *J* = 8.3), 7.93 (s, 1H), 7.91 (s, 1H), 7.58 (t, *J* = 8.0, 1H), 7.46–7.43 (m, 3H), 7.26 (s, 2H), 5.47 (s, 2H). ^13^C NMR (DMSO-d_6_): *δ* 165.5, 155.9, 152.6, 149.4, 140.8, 140.7, 139.8, 133.8, 129.8, 129.2 (d, *J* = 31.5), 128.0, 127.4, 124.0 (d, *J* = 272.1), 123.6, 119.8 (d, 2C), 118.6, 116.2 (d, 2C), 45.8; ^19^F NMR (DMSO-d_6_): *δ* −61.28; HRMS (M + H)^+^ calcd. for C_20_H_15_F_3_N_6_O: 412.1259, found 413.1324.

*4-((2,6-Dichloro-9H-purin-9-yl)methyl)-N-(3-(4-methyl-1H-imidazol-1-yl)-5-(trifluoromethyl)-phenyl)benzamide* (**10**): yield 84%, white solid, m.p. >350 °C. ^1^H NMR (DMSO-d_6_): *δ* 10.69 (s, 1H), 8.88 (s, 1H), 8.25 (s, 1H), 8.20 (s, 1H), 8.12 (s, 1H), 7.97 (s, 1H), 7.95 (s, 1H), 7.72 (s, 1H), 7.51–7.47 (m, 3H), 5.62 (s, 2H), 2.17 (s, 3H). ^13^C NMR (DMSO-d_6_): *δ* 165.5, 153.4, 151.1, 149.4, 141.1, 139.6, 138.8, 137.9, 134.9, 133.7, 130.8 (d, *J* = 32.1), 130.5, 128.2, 127.6, 123.6 (d, *J* = 272.2), 114.8, 114.2, 111.7, 46.7, 13.4; ^19^F NMR (DMSO-d_6_): −61.35. HRMS (M + H)^+^ calcd. for C_24_H_16_Cl_2_F_3_N_7_O: 545.0545, found 546.0812.

*4-((6-Amino-2-fluoro-9H-purin-9-yl)methyl)-N-(3-(4-methyl-1H-imidazol-1-yl)-5-(trifluoromethyl) phenyl)benzamide* (**11**): yield 68%, white solid, m.p. 230–232 °C. ^1^H NMR (DMSO-d_6_): *δ* 10.67 (s, 1H), 8.28 (s, 1H), 8.26 (s, 1H), 8.20 (d, 1H, *J* = 1.0), 8.11 (s, 1H), 7.97 (s, 1H), 7.95 (s, 1H), 7.84 (br.s, 2H), 7.72 (s, 1H), 7.47–7.45 (d, 3H), 5.43 (s, 2H), 2.18 (s, 3H). ^13^C NMR (DMSO-d_6_): *δ* 165.6, 158.5 (d, *J* = 254.8), 157.8 (d, *J* = 29.9), 150.7 (d, *J* = 20.1), 141.2, 141.1, 140.7, 138.8, 137.8, 134.8, 133.5, 130.7 (d, *J* = 32.3), 128.1, 127.4, 123.5 (d, *J* = 272.7), 117.0 (d, 2C), 114.8, 114.1, 111.6, 45.9, 13.4. ^19^F NMR (DMSO-d_6_): *δ* −61.36, −52.10. HRMS (M + H)^+^ calcd. for C_24_H_18_F_4_N_8_O: 510.4566, found 511.1605.

*4-((6-Amino-9H-purin-9-yl)methyl)-N-(3-(4-methyl-1H-imidazol-1-yl)-5-(trifluoromethyl)phenyl) benzamide* (**12**): yield 89%, white solid, m.p. 268–270 °C. ^1^H NMR (DMSO-d_6_): 10.66 (s, 1H), 8.31 (s, 1H), 8.25 (s, 1H), 8.20 (s, 1H), 8.15 (s, 1H), 8.11 (s, 1H), 7.95 (s, 1H), 7.93 (s, 1H), 7.72 (s, 1H), 7.47–7.46 (d, 3H), 7.27 (s, 2H), 5.48 (s, 2H), 2.17 (s, 3H) ppm. ^13^C NMR (DMSO-d_6_): *δ* 165.7, 156.0, 152.6, 149.4, 141.2, 141.1, 140.8, 138.8, 137.9, 134.9, 133.4, 130.8 (d, *J* = 32.3), 128.1, 127.5, 123.6 (d, *J* = 273.0), 118.7, 114.8, 114.2, 111.6 (d, 2C), 45.9, 13.5. ^19^F NMR (DMSO-d_6_): *δ* −61.35; HRMS (M + H)^+^ calcd. for C_24_H_19_F_3_N_8_O: 492.1634, found 493.1695.

#### 3.1.3. General Method for the Synthesis of Key Compounds **13** and **14**

To a solution of **7** or **10** in dry methanol, K_2_CO_3_ was added. The reaction mixture was stirred for 30 min at 50 °C, then brought to room temperature and added water. Methanol was evaporated under vacuum. The obtained residue was extracted by CHCl_3_ three times. The combined organic layers were dried over Na_2_SO_4_, filtered and concentrated under vacuum. The product was purified by column chromatography on silica gel. The fractions containing the products **13** or **12** were collected and evaporated to dryness under vacuum. The residue was crystallized.

*4-((2-Chloro-6-methoxy-9H-purin-9-yl)methyl)-N-(3-(trifluoromethyl)phenyl)benzamide* (**13**): yield 69%, white solid, m.p. 212–214 °C. ^1^H NMR (DMSO-d_6_): *δ* 10.53 (s, 1H), 8.58 (s, 1H), 8.23 (s, 1H), 8.01 (d, 1H, *J* = 8.5), 7.95 (s, 1H), 7.93 (s, 1H), 7.59 (t, 1H, *J* = 8.0), 7.47–7.43 (d, 3H), 5.56 (s, 2H), 4.11 (s, 3H). ^13^C NMR (DMSO-d_6_): *δ* 165.4, 160.8, 153.1, 151.5, 144.5, 139.8, 139.7, 133.9, 129.2 (d, *J* = 31.9), 128.1, 127.3, 123.6 (d, *J* = 272.6), 119.8, 116.1 (d, 2C), 54.9, 43.6. ^19^F NMR (DMSO-d_6_): −61.29. HRMS (M + H)^+^ calcd. for C_21_H_15_ClF_3_N_5_O_2_: 461.0866, found 462.0929.

*4-((2-Chloro-6-methoxy-9H-purin-9-yl)methyl)-N-(3-(4-methyl-1H-imidazol-1-yl)-5-(trifluoromethyl)phenyl)benzamide* (**14**): yield 45%, white solid, m.p. 160–162 °C. ^1^H NMR (DMSO-d_6_): *δ* 10.67 (s, 1H), 8.58 (s, 1H), 8.26 (s, 1H), 8.19 (s, 1H), 8.11(s, 1H), 7.97 (s, 1H), 7.95 (s, 1H), 7.72 (s, 1H), 7.47–7.45 (d, 3H), 5.56 (s, 2H), 4.11 (s, 3H), 2.17 (s, 3H). ^13^C NMR (DMSO-d_6_): *δ* 165.5, 160.8, 153.1, 151.5, 144.5, 141.1, 140.1, 138.8, 137.8, 134.8, 133.6, 130.7 (d, *J* = 32.3), 128.2, 127.3, 123.5 (d, *J* = 272.6), 119.8, 114.7, 114.1, 111.6, 54.9, 43.6, 13.4. ^19^F NMR (DMSO-d_6_): −61.36; HRMS (M + H)^+^ calcd. for C_25_H_19_ClF_3_N_7_O_2_: 541.1241, found 542.1310.

#### 3.1.4. General Method for the Synthesis of Key Compounds **15** and **16**

To a solution of **7** or **10** in dry acetonitrile, 25% aq. NH_4_OH was added dropwise. The reaction mixture was stirred for 1 h at 50 °C, then brought to room temperature and water added. Acetonitrile was evaporated under vacuum. The obtained residue was extracted by CHCl_3_ three times. The combined organic layers were dried over Na_2_SO_4_, filtered, and concentrated under vacuum. The product was purified by column chromatography on silica gel. The fractions containing the products **15** or **16** were collected and evaporated to dryness under vacuum. The residue was crystallized.

*4-((6-Amino-2-chloro-9H-purin-9-yl)methyl)-N-(3-(trifluoromethyl)phenyl)benzamide* (**15**): yield 83%, white solid, m.p. 272–274 °C. ^1^H NMR (DMSO-d_6_): *δ* 10.53 (s, 1H), 8.30 (s, 1H), 8.22 (s, 1H), 8.01 (d, 1H, *J*=8.3 Hz), 7.95 (s, 1H), 7.93 (s, 1H), 7.82 (br.s, 2H), 7.59 (t, 1H, *J* = 8.0), 7.45–7.41 (m, 3H), 5.45 (s, 2H). ^13^C NMR (DMSO-d_6_): *δ* 165.5, 156.8, 153.1, 150.5, 141.5, 140.4, 139.8, 133.9, 129.8, 129.3 (d, *J* = 31.4), 128.2, 127.2, 124.1 (d, *J* = 272.5), 123.6, 119.9 (d, 2C), 117.7, 116.2 (d, 2C), 45.9. ^19^F NMR (DMSO-d_6_): *δ* −61.27; HRMS (M + H)^+^ calcd. for C_20_H_14_F_3_N_6_O: 446.0870, found 447.0933.

*4-((6-Amino-2-chloro-9H-purin-9-yl)methyl)-N-(3-(4-methyl-1H-imidazol-1-yl)-5-(trifluoromethyl) phenyl)benzamide* (**16**): yield 76%, white solid, m.p. 252–254 °C. ^1^H NMR (DMSO-d_6_): *δ* 10.67 (s, 1H), 8.31 (s, 1H), 8.27 (s, 1H), 8.20 (d, 1H, *J* = 1.3), 8.11 (s, 1H), 7.97 (s, 1H), 7.95 (s, 1H), 7.82 (br.s, 2H), 7.72 (s, 1H), 7.47 (s, 1H), 7.45 (s, 1H), 7.43 (s, 1H), 5.46 (s, 2H), 2.18 (s, 3H). ^13^C NMR (DMSO-d_6_): *δ* 165.6, 156.7, 153.1, 150.5, 141.4, 141.1, 140.7, 138.8, 137.8, 134.9, 133.5, 130.7 (d, *J* = 32.3), 128.1, 127.3, 123.5 (d, *J* = 272.6), 117.7, 114.8, 114.1, 111.6 (d, 2C), 45.9, 13.4. ^19^F NMR (DMSO-d_6_): −61.36. HRMS (M + H)^+^ calcd. for C_24_H_18_ClF_3_N_8_O: 526.1244, found 527.1316.

### 3.2. Cytotoxicity Assay

Each in vitro experiment was performed at least in triplicate and the standard deviation of absorbance was less than 10% of the mean. For the in vitro assays, a stock solution (1% DMSO in the appropriate buffer with the tested compound diluted under sonication) was prepared from which several dilutions were made with the appropriate buffer.

Human cell lines were obtained from the Institute of Cytology, Russian Academy of Sciences. The cell lines studied were maintained in RPMI 1640 medium (AppliChem, Darmstadt, Germany) supplemented with 20% (HL-60, RPMI 1788) fetal calf serum (HyClone, Cramlington, UK), in DMEM (A 549) and Eagle’s MEM (AppliChem) (MCF7, HeLa) with 10% fetal calf serum (HyClone). Cells were cultivated at 37 °C in a humidified atmosphere of 5% CO_2_. Cellular sensitivities to synthesized compounds and standards were measured with the MTT assay. Cells were plated in triplicate in 96-well plates (105 cells/well for suspended cell cultures and 5 × 103 cells/well for monolayer cultures); on the same day (suspended cell cultures) or on the following day (monolayer cultures), compounds were added at the appropriate dilutions. Anti-proliferative activity was studied at concentrations 0.1–50.0 µM. The reference drugs were imatinib, sorafenib and nilotinib. Plates were incubated under standard conditions for 48 h. Thereafter, 10 μL MTT (Sigma) in phosphate-buffered saline (5 mg/mL) was added. The plates were incubated for an additional 4 h and 150 μL dimethylsulphoxide (DMSO) was added to dissolve the formazan crystals. The optical density was read on a HALO MPR-95 Microplate Reader (Dynamica Scientific Ltd., Melbourne, Victoria, Australia) at 570 nm. The antiproliferative effects were taken as degrees of inhibition of tumor cells (%). The average of three parallel measurements was calculated for each concentration of test compound. The degree of cell growth suppression was plotted as a function of the logarithm of the concentration. The half maximal inhibitory concentration (IC_50_) was determined graphically for active compounds.

### 3.3. Kinase Inhibitory Assay

#### 3.3.1. Abl1 Kinase Inhibitory Assay

The Abl1 inhibitory activity assay was performed using Abl1 Kinase Enzyme System (Promega, Madison, WI, USA) and ADP-Glo™ Kinase assay kit (Promega, Madison, WI, USA) according to the Technical Manual. The general procedure was as follows: kinase (2.5 ng/reaction) was incubated with substrate (1 μg), compound (0.5 and 10µM) and ATP (50 μM) in a commercial buffer solution with a reaction volume of 5 μL. In every experiment, no-enzyme and no-compound control reactions were included to represent background luminescence (0% activity) and uninhibited kinase activity (100% activity), respectively. The assay 384-well plate was incubated at room temperature for 1 h. Afterwards, 5μL of ADP-Glo reagent was added into each well to stop the reaction and consume the remaining ADP within 40 min. At the end, 10 μL of kinase detection reagent was added into the well and incubated for 30 min to produce a luminescence signal. Kinase activity assays were performed in triplicate at each inhibitor concentration. The luminescence was measured with the help of a Tecan Infinite M200 Plate Reader. The luminescence data were analyzed using the computer software, Magellan™. Percent kinase activity (KA) was calculated by subtracting the average no-enzyme control luminescence values from all kinase-containing reactions with or without compound, then converting these net luminescence values to percent activity based on the no-compound control reactions representing 100% kinase activity. Inhibition (I, %) was calculated as 100%-KA.

#### 3.3.2. Inhibitory Assay on the Panel of Tyrosine Kinases

The single-dose screening of kinase selectivity was performed using Kinase Selectivity Profiling System TK-1 (Promega, Madison, WI, USA) and ADP-Glo™ Kinase assay kit (Promega, Madison, WI, USA) according to Technical Manual. Kinase Selectivity Profiling System TK-1 includes a set of kinases from the tyrosine kinase family (EGFR, HER2, HER4, IGF1R, InsR, KDR, PDGFα, PDGFβ) in 8-tube strip format. The general procedure was as follow: kinase working stock (2 µL/reaction) was incubated with ATP/substrate working stock (1 µL) and compound (1 µM) in a commercial buffer solution with a reaction volume of 5 μL. The reaction conditions and inhibition (%) calculation were the same as in Section 3.3.1. Kinase activity assays were performed in duplicate at each inhibitor.

### 3.4. Cell Apoptosis Assay

Tumor cells were seeded on a 6-well plate at a density of 200.000 per well for HL-60 and 100.000 per well for OKP-GS. Test compounds **7** and **10** were added at concentrations of 5, 10, and 20 μM for 72 h. Then, the cells were washed from the culture medium by centrifugation at 1000 rpm for 5 min, washed again with PBS, then fixed with ice-cold ethanol during constant shaking and incubated at −20 °C for 24 h. After that, we washed it from alcohol by centrifugation, washed it again with PBS and added a solution containing 100 μg/mL RNase A and 10 μg/mL propidium iodide (PI), incubated for 40 min in the dark and measure it on a Cytomics FC500 Beckman Coulter flow cytometer (FL4 channel for PI). Received data were analyzed with the help of Kaluza 2.0 Software (Beckman Coulter, Brea, CA, USA).

### 3.5. In Vitro Cell Cycle Effects

Tumor cells were plated on a 6-well plate at a density of 100.000 per well for OKP-GS. Test compounds **7** and **10** were added at concentrations of 5, 10, and 20 μM for 72 h. Then, the cells were washed from the culture medium by centrifugation at 1000 rpm for 5 min, washed again with phosphate buffer, then the Annexin V-FITC and PI dyes from the Annexin 5A-FITC Kit (Beckman Coulter, Brea, CA, USA) were added, according to the manufacturer’s instructions. Colored suspensions were incubated for 15 min in the dark on ice and measured on a Cytomics FC500 Beckman Coulter flow cytometer (channels FL1 for Annexin V-FITC and FL4 for PI). Data were analyzed with Kaluza 2.0 Software (Beckman Coulter, Brea, CA, USA).

### 3.6. Molecular Docking

AutoDock Vina was used for docking studies [24]. Two-dimensional structures were created using Marvin Sketch [33]. Three-dimensional structures were generated using molconvert [34]. Chemical file format conversions were performed with Open Babel [36]. Missing protein residues were restored with MODELLER [37]. Preparation of ligands and receptors for docking was carried out in MGL Tools [38]. Visualization of docking results, H-bond search and structure minimizations were made using Chimera [39]. Two-dimensional interactions maps were generated by PoseView [49]. The most informative snapshots of molecular dynamics trajectory were used for H-bond location and interaction map creation.

### 3.7. Molecular Dynamics Simulations

Molecular dynamics were carried out using GROMACS package using AMBER FF99SB-ILDN force field. All ligands were parametrized with ACPYPE [38]. Simulation workflow was as follows: solvation, neutralizing and adding NaCl ions up to concentration of 0.15 mol, energy minimization, NPT and NVT equilibration steps of 200 ps each and a final 2 ns production run at 300 K. A dodecahedron box of 1.2 nm and periodic bounding conditions were used. A Berendsen thermostat was used for equilibration. Long electrostatic coupling was treated according to PME method. G_mmpbsa tool was used for MM-PBSA binding energy calculations [26]. To perform energy calculations, every twentieth frame of the final molecular dynamics trajectory was extracted, skipping the first 100 frames.

## 4. Conclusions

As part of our study, a series of new (arylaminomethyl) benzamide derivatives containing substituted purines as promising fragments to enforce the binding to a kinase’s hinge region were developed and studied for their antitumor activity.

Compounds **7** and **10**, containing chlorine atoms in the C-2 and C-6 positions of the purine heterocycle, demonstrated the highest activity against all studied cancer cell lines, comparable to the data for sorafenib. The highest activity was observed against the leukemic cell line K562 with IC_50_ values of 2.27 µM and 2.53 µM, and IC_50_ values for HL-60 equal to 1.42 and 1.52 µM, respectively. The absence of a 4-methyl-imidazole fragment in structures **7** and **13** did not have a noticeable effect on their antiproliferative activity in comparison with analogs **10** and **14**. Several synthesized purine derivatives were also able to inhibit platelet-derived growth factor (PDGF) in vitro at a concentration of 1 µM with a best value of 45% for compound **9**.

Compounds **7** and **10** were found to induce apoptosis induction in a dose-dependent manner and, at a concentration of 20 μM, the compounds exhibited a more than two-fold higher level of apoptosis compared with sorafnib. In cell cycle progression studies, compounds **7** and **10** decreased the proliferation of OKP-GS cells. The effect was more significant than that of sorafenib at 20 μM. Compound **7** dose-dependently delayed cells in the G2/M phase.

The results of docking and molecular dynamics show that the presence of a 4-methylimidazole fragment in the structure of titled compounds promotes their binding to protein kinases predominantly as type 2 inhibitors. However, in the case of Aurora kinases, one type of binding prevailed, regardless of the presence of a 4-methylimidazole fragment.

Thus, substituted purines in the form of a hinge-binding moiety in combination with a flexible linker, showing promising enzymatic inhibition as well as antiproliferative activity, can be considered a valuable starting point for further studies.

## Figures and Tables

**Table 1:** In vitro anti-proliferative activities of target compounds **7**–**16** against cancer cell lines (K562, HL-60, MCF-7, Hela, HepG2, A-549, OKP-GS) and normal human lymphoblastoid cells (RPMI 1788).

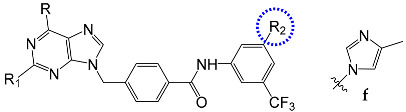											
Comp.	R	R_1_	R_2_	Anti-Proliferative Activities in Deferent Cancer Cell Lines(IC_50_ µM) ^1^							Inhibition, at 100 µM, %
				K562	HL-60	MCF-7	HeLa	HepG2	A549	OKP-GS	RPMI 1788
**7**	Cl	Cl	H	2.27 ± 0.81	1.42 ± 0.54	12.35 ± 4.07	6.93 ± 1.18	11.29 ± 1.44	26.95± 0.64	4.56 ± 1.73	98.89
**8**	NH_2_	F	H	n/a ^2^	>50	n/a	n/a	n/a	n/a	n/a	33.49
**9**	NH_2_	H	H	n/a	n/a	n/a	n/a	n/a	n/a	n/a	56.75
**10**	Cl	Cl	**f**	2.53 ± 0.77	1.52 ± 0.66	8.10 ± 1.98	6.05 ± 1.03	12.72 ± 0.70	17.96 ± 0.73	24.77 ± 3.25	98.53
**11**	NH_2_	F	**f**	n/a	29.67 ± 1.19	n/a	n/a	n/a	n/a	n/a	93.41
**12**	NH_2_	H	**f**	n/a	n/a	n/a	n/a	n/a	n/a	n/a	n/a
**13**	OMe	Cl	H	29.68 ± 1.87	26.11 ± 1.23	14.23 ± 2.71	15.43 ± 1.92	9.16 ± 0.78	12.95 ± 0.67	15.53 ± 2.27	100.00
**14**	OMe	Cl	**f**	27.46 ± 1.19	48.56 ± 3.02	67.41 ± 3.29	33.73 ± 7.49	28.50 ± 1.07	20.11 ± 0.81	10.63 ± 2.88	99.09
**15**	NH_2_	Cl	H	30.60 ± 0.94	7.18	76.77 ± 3.25	n/a	88.29 ± 2.54	n/a	n/a	93.52
**16**	NH_2_	Cl	**f**	n/a	n/a	n/a	n/a	n/a	n/a	n/a	5.91
**Reference Compounds**											
Imatinib				0.06 ± 0.03	10.23	n/d ^3^	n/d	n/d	n/d	n/d	98.95
Sorafenib				2.90 ± 0.4	n/d	13.67 ± 3.08	6.82 ± 2.64	8.40 ± 2.80	6.01 ± 1.27	13.61 ± 1.71	100.00
Nilotinib				<0.1	55	n/d	n/d	n/d	n/d	55	71.00

^1^ IC_50_—the concentration that causes 50% cell growth inhibition; ^2^ n/a—not applicable, ^3^ n/d—no data.

**Table 2:** Inhibition of Bcr–Abl1 kinases in the presence of compound **7**–**16** using ADP-Glo assay method.

	Compounds *												
	7	8	9	10	11	12	13	14	15	16	Imt	Nil	Sor
% Inhibition at 0.5 µM	24	23	39	36	37	38	24	18	17	15	58	86	23
% Inhibition at 10 µM	16	34	34	43	37	14	21	17	28	36	81	100	62

* Imt—imatinib; Nil—nilotinib; Sor—sorafenib.

**Table 3:** Inhibition of receptor tyrosine kinases by compounds **7**–**16**, sorafenib and lapatinib.

% Inhibition at 1 µM												
Kinases	Lapatinib	Sorafenib	7	8	9	10	11	12	13	14	15	16
EGFR	100	0	6	15	11	9	14	17	29	9	0	13
HER2	68	0	22	26	0	11	0	0	0	8	0	0
HER4	94	0	0	23	0	0	0	0	0	0	0	0
IGF1R	0	0	0	5	0	0	0	0	0	0	0	0
InsR	0	0	6	0	0	0	0	0	0	17	0	0
KDR	17	90	11	14	2	16	12	0	30	27	13	12
PDGFRα	4	98	40	13	45	37	4	22	10	16	16	17
PDGFRβ	7	95	30	32	36	39	0	36	17	20	33	6

**Table 4:** Distribution of OKP-GS cells by phases of the cell cycle under the influence of compounds **7** and **10** at 72 h of exposure.

Cell Cycle Phase	Control OKP-GS	Concentration						
		Comp. 7			Comp. 10			Sorafenib
		5 μM	10 μM	20 μM	5 μM	10 μM	20 μM	20 μM
G1, %	63.08	60.9	55.5	51.2	56.9	53.3	53,2	61.9
S, %	14.95	14.2	17.7	17.4	16.8	21.5	19.3	17.3
G2/M, %	20.43	23.5	26.0	29.9	24.3	24.2	25.3	19.7

**Table 5:** AutoDock Vina’s docking scores (kcal/mol) for the target (**7**–**16**) and reference compounds.

Kinase	PDB	7	8	9	10	11	12	13	14	15	16	Native	Native, min
Abl	2HYY	−10.3	−10.8	−10.6	−9.8	−10.8	−10.9	−10.2	−9.9	−10.6	−9.7	−12.7 *	−12.5 *
Abl	3CS9	−10.2	−10.4	−10.5	−10.4	−11.7	−11.7	−9.7	−10.5	−9.8	−10.3	−13.6 *	−11.6 *
LCK	2PL0	−9.5	−9.2	−9.3	−7.5	−9.1	−9.7	−9.3	−7.5	−9.1	−8.3	−10.6 *	−10.6 *
PARP	4TVJ	−10.7	−10.7	−10.8	−11.6	−11.7	−11.6	−10.8	−11.4	−10.7	−11.4	−13.6	−13.6
PARP	7KK4	−10.6	−10.4	−10.3	−10.6	−10.9	−10.9	−10.3	−10.3	−10.2	−10.4	−14.2	−13.6
c-Met	1R0P	−8.9	−8.5	−8.6	−9.9	−9.7	−9.6	−8.6	−9.5	−8.9	−9.6	−9.0	n/d
P38MAP/Sor	3GCS	−9.3	−9.7	−7.7	−10.0	−9.7	−8.2	−8.3	−7.6	−7.7	−9.6	−10.7 *	−10.9 *
KDR/Sor	3WZE	−9.7	−10.2	−9.9	−5.6	−6.7	−6.9	−9.1	−3.4	−10.1	−7.0	−12.3 *	−11.5 *
BRAF/cop	5HI2	−10	−10.7	−10.5	−2.4	−3.9	−4.8	−10.1	−1.1	−10.5	−3.2	−11.2 *	−11 *
BRaf V600E	3OG7	−10.1	−10.7	−10.9	−9.6	−9.1	−9.4	−10.6	−8.6	−10.7	−9.3	−11.5	−12
Wee1	2IN6	−9.8	−9.1	−9.8	−7.3	−9.3	−8.7	−9.6	−7.9	−9.0	−8.4	−11.9	−12
ROS1	3ZBF	−7.0	−7.6	−7.0	−6.8	−6.9	−7.1	−7.2	−7.0	−7.2	−6.8	−8.5	−8.8
HER2	3PP0	−12.0	−12.1	−11.6	−9.9	−10.8	−10.4	−11.7	−9.4	−12.0	−10.8	−11.4	−11.5
ErbB4	3BBT	−9.8	−10.8	−10.5	−8.7	−9.3	−8.8	−10.2	−7.9	−9.8	−9.1	−10.1	−10.2
VEGFR1	3HNG	−11.5	−11.7	−11.5	−9.4	−10.4	−10.6	−10.5	−9.3	−11.8	−9.9	−11	−11
VEGFR2	4AG8	−9.5	−10.6	−10.2	−11.6	−10.7	−10.5	−10.6	−8.8	−10.4	−11.4	−12.6	−12.6
VEGFR2	4ASD	−9.4	−9.9	−9.8	−5.9	−6.7	−5.3	−9.0	−2.9	−9.7	−7.0	n/d **	n/d
EGFR T790M	4G5P	−9.4	−9.1	−8.8	−9.7	−9.0	−8.9	−9.2	−9.5	−9.2	−8.9	−8.4	−8.5
RET	6NEC	−9.3	−9.4	−9.3	−10.2	−9.8	−9.8	−9.0	−9.8	−9.4	−9.9	−11.5	−10.8
TRKB	4AT3	−10.7	−11.4	−11.2	−8.7	−6.2	−8.9	−10.1	−7.8	−11.3	−9.0	−11.2	−11.2
ALK	4DCE	−8.1	−8.1	−8.2	−8.8	−8.6	−8.8	−8.1	−8.3	−8.1	−8.5	−11.1	n/d
MEK1	4LMN	−9.8	−10.0	−9.7	−10.6	−10.3	−10.0	−9.7	−10.2	−10.1	−10.4	−11.5	−11.1
BTK	5KUP	−10.6	−10.6	−10.2	−10.4	−10.5	−10.4	−9.8	−10.4	−10.6	−10.2	−12.6	−12.5
TRKA	5KVT	−9.9	−9.9	−10.2	−10.8	−10.4	−10.3	−9.8	−10.7	−9.7	−10.4	−12.1	−11.9
JAK3	5TOZ	−7.1	−7.8	−7.6	−5.3	−5.3	−5.6	−6.8	−5.4	−7.8	−4.8	−7.5	−7.5
SYK	5Y5U	−8.3	−8.6	−7.4	−8.6	−8.2	−7.9	−8.2	−4.9	−8.1	−7.4	−9.5	−9.6
TRKc	6KZD	−12.3	−12.4	−11.8	−13.7	−13.2	−12.9	−12.3	−13.3	−12.4	−13.3	−15.3	−15.2
Aurora A	5EW9	−9.3	−9.2	−9.1	−9.8	−9.6	−9.2	−9.1	−9.3	−9.2	−9.7	−9.7	−9.7
Aurora B	2BFY	−9.5	−9.2	−9.0	−10.4	−10.2	−9.9	−9.3	−10.2	−9.2	−10.1	−8.5	−9.7
Aurora B	2VRX	−9.8	−9.6	−9.6	−9.7	−9.7	−9.3	−9.4	−9.2	−9.4	−9.6	−9.6	−9.7
Aurora B	4B8M	−9.4	−9.1	−8.9	−10.0	−9.5	−9.4	−9.3	−9.5	−9.2	−9.6	−8.9	n/d
Aurora B	4C2W	−9.3	−8.9	−8.7	−9.5	−9.3	−9.1	−9.0	−9.3	−8.9	−9.3	−7.1	−7.5

* Data from previous studies [25]; ** n/d—did not define.

**Table 6:** Calculated binding affinities for complexes of target structures with protein kinases and reference compounds.

Kinase	PDB	LIGAND	VDW	EL	SOLV	TOTAL
Abl	3CS9	**10**	−280.604+/−13.300	−33.748+/−10.149	197.295+/−18.125	−144.216+/−13.887
Abl	3CS9	**11**	−269.266+/−11.421	−23.543+/−12.111	192.086+/−15.869	−127.050+/−15.142
Abl	3CS9	**12**	−273.217+/−11.025	−35.440+/−10.634	196.182+/−16.454	−137.285+/−16.592
BRAF	5HI2	**8**	−205.424+/−15.037	−56.280+/−11.941	187.970+/−13.844	−95.507+/−15.838
BRAF	5HI2	**9**	−209.510+/−11.641	−48.733+/−7.868	204.317+/−13.430	−75.479+/−13.532
PARP	4TVJ	**12**	−252.293+/−9.330	−22.457+/−7.934	243.052+/−13.752	−57.159+/−12.996
PARP	4TVJ	(1*)	−254.429+/−12.402	−80.820+/−10.301	285.878+/−18.010	−75.431+/−17.052
TRKc	6KZD	**10**	−267.547+/−13.579	−30.315+/−14.399	190.950+/−26.540	−132.853+/−17.438
TRKc	6KZD	**16**	−260.400+/−14.121	−37.796+/−12.815	225.936+/−24.329	−98.854+/−15.411
TRKc	6KZD	(2*)	−345.481+/−12.568	−66.215+/−11.188	237.491+/−15.570	−206.913+/−15.208
VEGFR1	3HNG	**15**	−221.420+/−11.969	−53.584+/−10.367	201.622+/−13.155	−94.144+/−12.719
PDGFRα	6JOL	**7**	−218.500+/−12.040	−36.081+/−10.847	164.907+/−20.821	−109.837+/−12.601
PDGFRβ	6JOL	**10**	−258.889+/−14.800	−39.871+/−7.778	190.59+/−20.236	−133.473+/−21.632
HER2	3PP0	**8**	−195.535+/−11.385	−21.103+/−11.925	164.059+/−14.272	−72.710+/−15.445
HER2	3PP0	(3*)	−297.072+/−19.550	−41.086+/−9.278	222.538+/−19.287	−143.553+/−21.942
ErbB4	3BBT	**8**	−198.066+/−14.896	−37.284+/−20.740	187.266+/−21.197	−70.008+/−18.625
TRKA	5KVT	**11**	−224.032+/−11.607	−39.853+/−12.923	209.776+/−23.192	−78.144+/−15.120
Aurora B	2BFY	**10**	−200.130+/−11.245	−39.298+/−12.353	176.014+/−20.661	−85.912+/−14.243
Aurora B	2BFY	**11**	−191.664+/−13.887	−48.587+/−22.055	206.697+/−27.319	−56.133+/−14.511
Aurora B	2BFY	**14**	−218.912+/−11.524	−52.308+/−10.948	220.736+/−17.088	−74.833+/−15.533
Aurora B	2BFY	**16**	−195.485+/−11.824	−36.962+/−14.128	190.842+/−24.456	−64.401+/−16.072

(1*) 4-(Chloromethyl)-N-(3-(4-methyl-1H-imidazol-1-yl)-5-(trifluoromethyl)phenyl)benzamide [25]; (2*) 3-((6-(4-aminophenyl)imidazo[1,2-a]pyrazin-3-yl)ethynyl)-N-(3-isopropyl-5-((4-methylpiperazin-1-yl)methyl)phenyl)-2-methylbenzamide [37]; (3*) 2-{2-[4-({5-chloro-6-[3-(trifluoromethyl)phenoxy]pyridin-3-yl}amino)-5H-pyrrolo[3,2-D]pyrimidin-5-yl]ethoxy}ethanol [40].

## Data Availability

Not applicable.

## Supplementary Materials

The following are available online at https://www.mdpi.com/article/10.3390/ijms222312738/s1.
