# Supplementary material for: Synthesis, In Vitro and In Silico Anticancer Activity of New 4-Methylbenzamide Derivatives Containing 2,6-Substituted Purines as Potential Protein Kinases Inhibitors
Source: Int J Mol Sci. 2021 Nov 25;22(23):12738. doi: 10.3390/ijms222312738 (PMC8657793; doi:10.3390/ijms222312738)
Supplement: Supplementary file 1 [file ijms-22-12738-s001.zip › Supplementary Material.pdf]

International Journal of Molecular Sciences

MDPI

Article

Synthesis, *In Vitro* and *In Silico* Anticancer Activity of New 4-Methylbenzamide Derivatives Containing 2,6-Substituted Purines as Potential Protein Kinases Inhibitors

Elena Kalinichenko [1]*, Aliaksandr Faryna [1], Tatyana Bozhok [1], Alesya Panibrat [1]

[1] Institute of Bioorganic Chemistry, National Academy of Sciences of Belarus, BY-220141, 5/2 Academician V.F. Kuprevich Street, Minsk, Belarus; info@iboch.by
* Correspondence: kalinichenko@iboch.by

Spectral data

^{1}H, ^{13}C and ^{19}F NMR spectra of compound **7** (DMSO-d6),

4-((2,6-dichloro-9H-purin-9-yl)methyl)-N-(3-(trifluoromethyl)phenyl)benzamide:

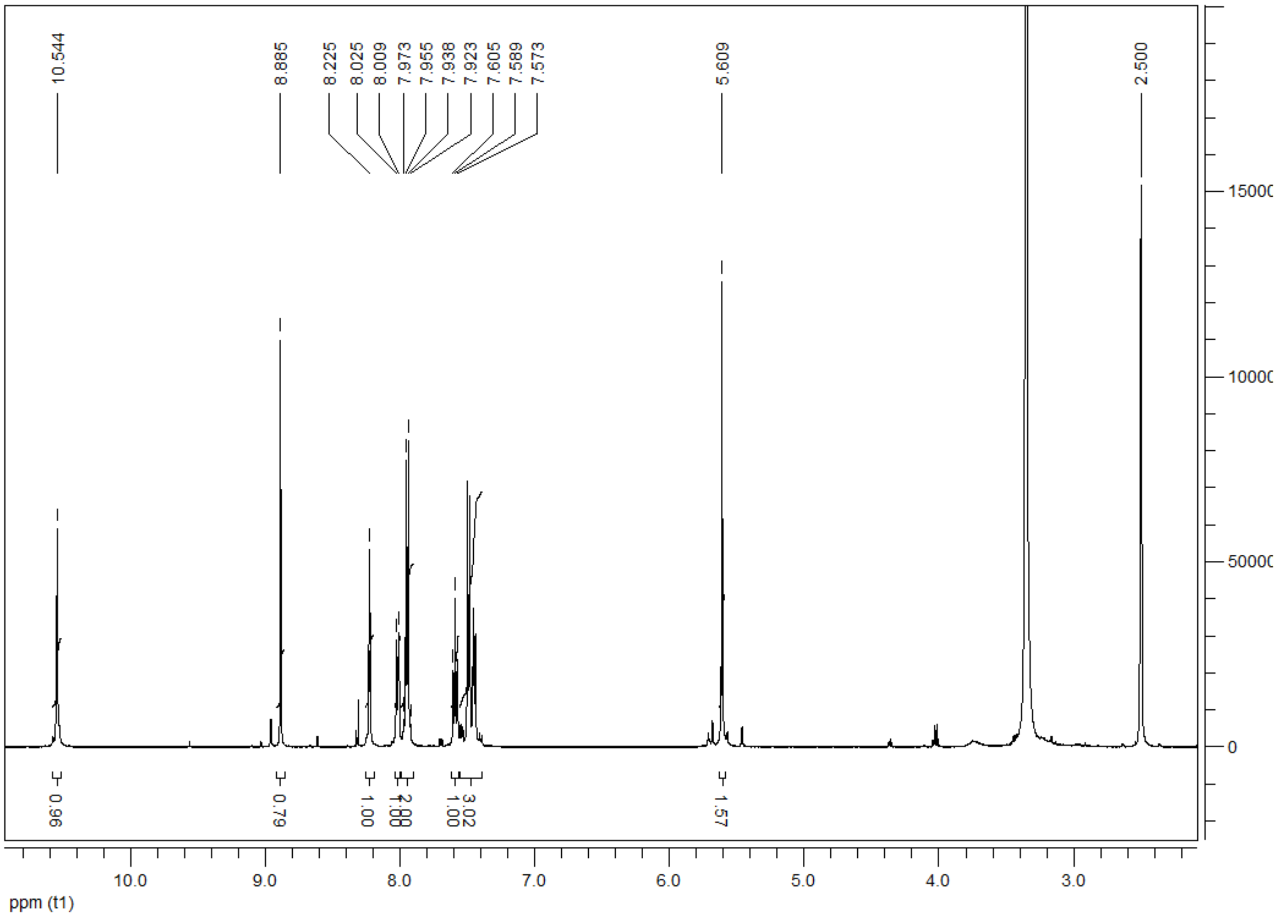

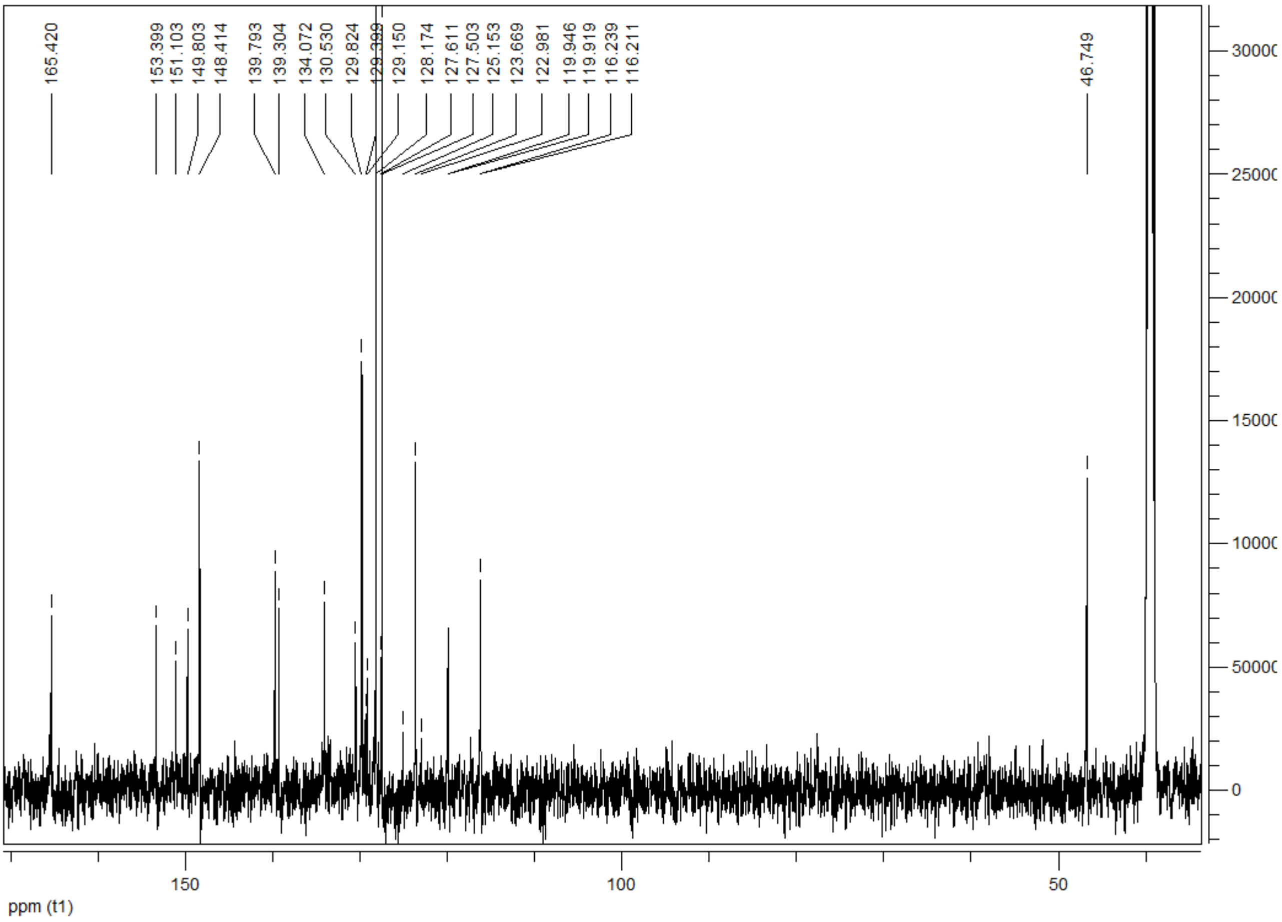

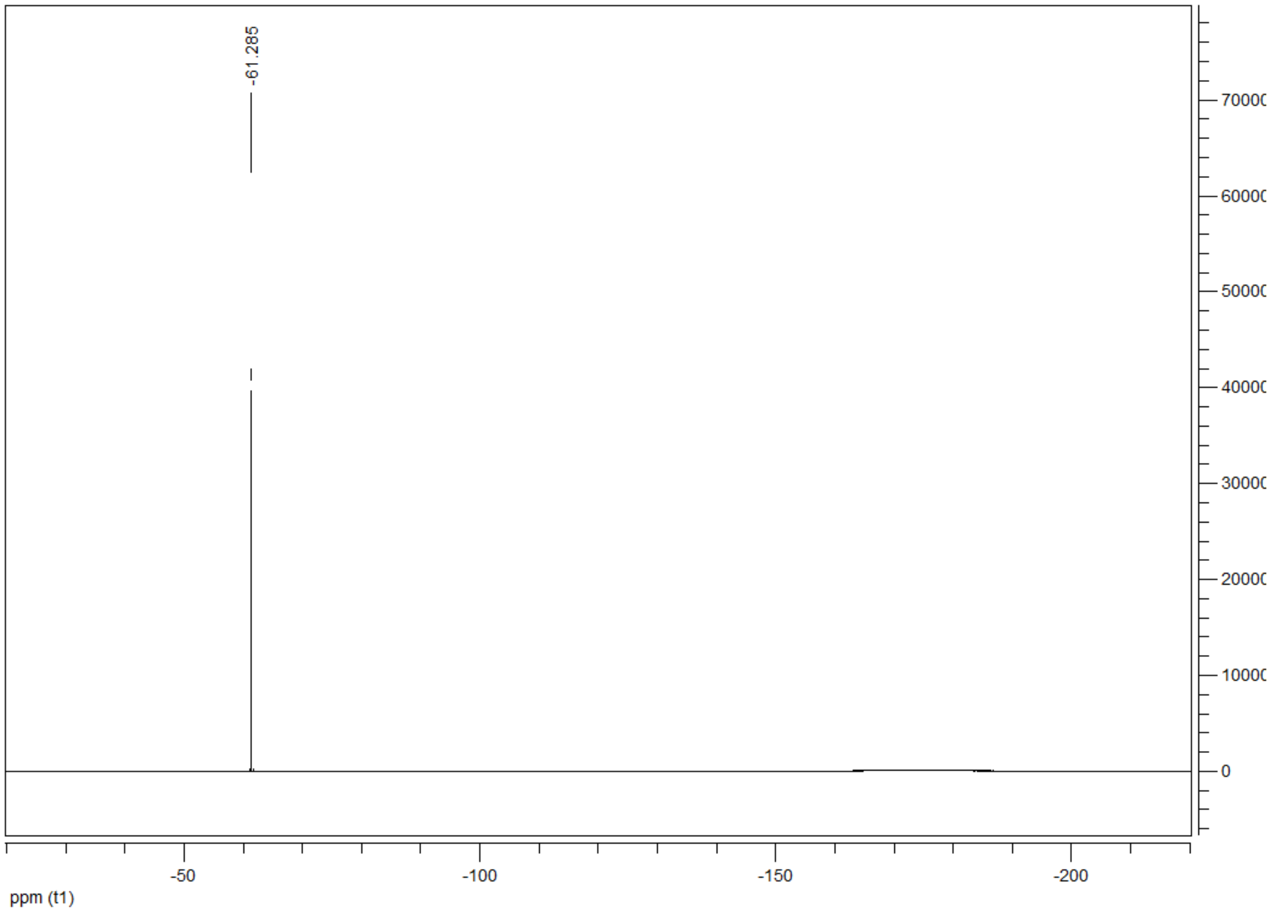

^{1}H, ^{13}C and ^{19}F NMR spectra of compound **8** (DMSO-d6),

4-((6-amino-2-fluoro-9H-purin-9-yl)methyl)-N-(3-(trifluoromethyl)phenyl)benzamide:

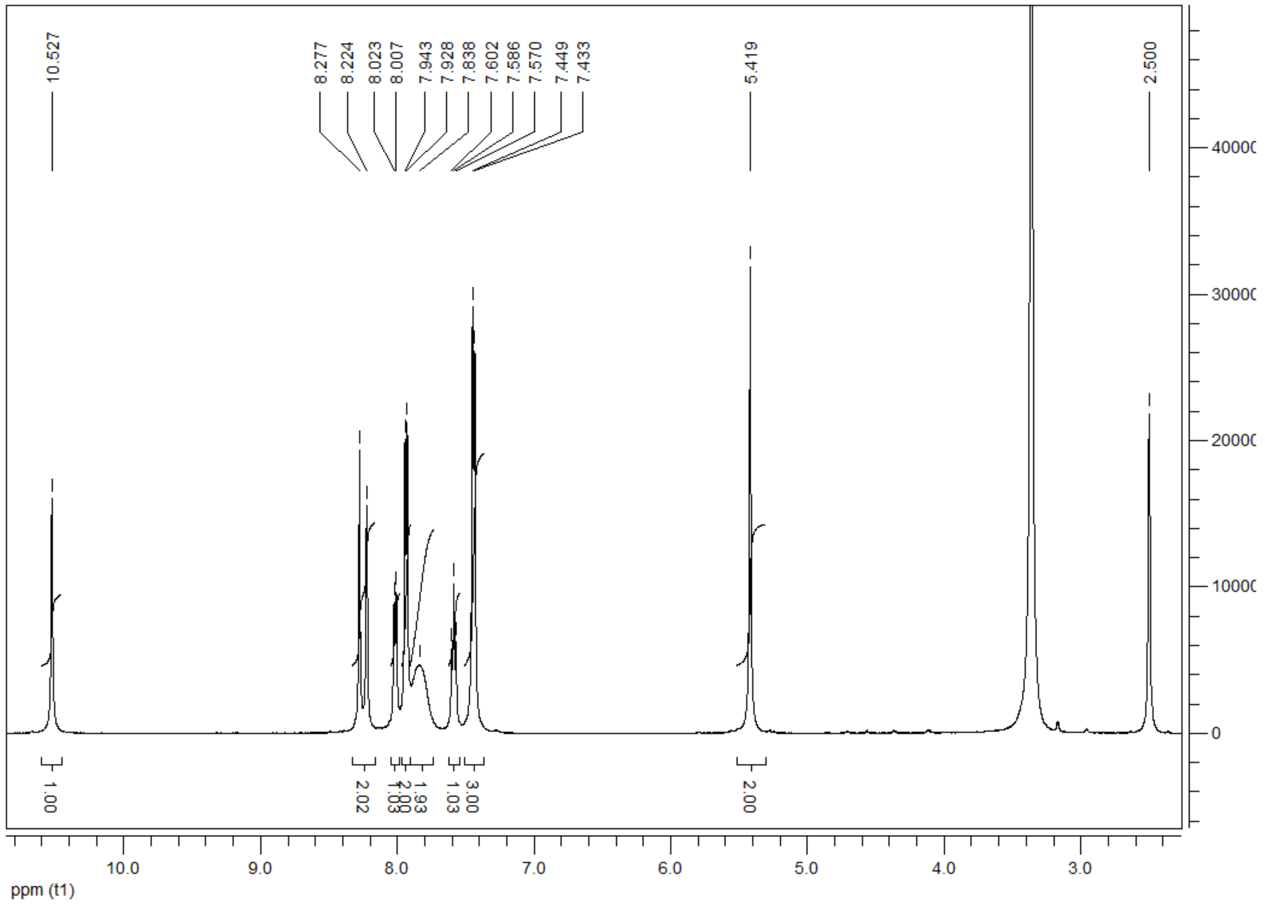

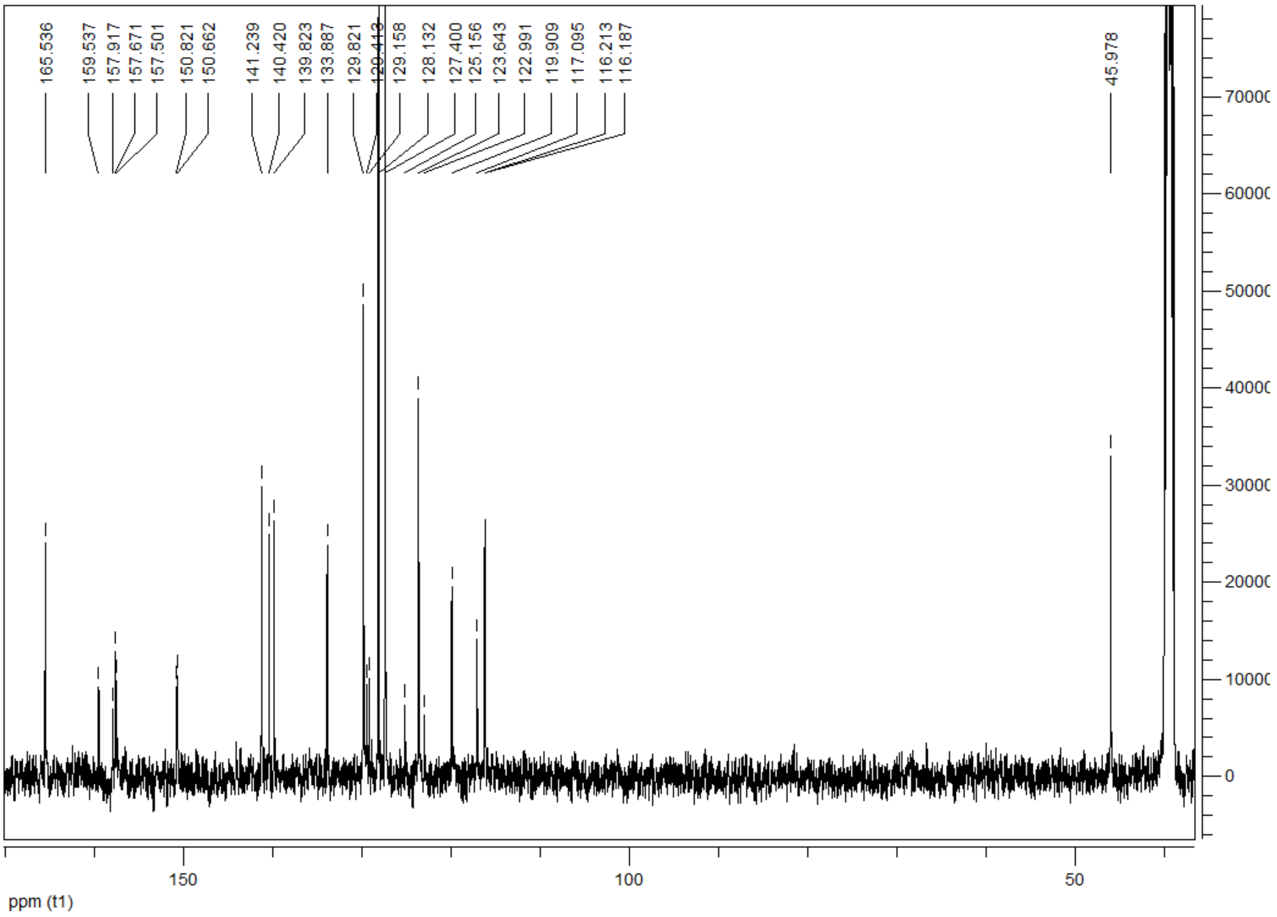
165.536
159.537
157.917
157.671
157.501
150.821
150.662
141.239
140.420
139.823
133.887
129.821
129.413
129.158
128.132
127.400
125.156
123.643
122.991
119.909
117.095
116.213
116.187
45.978
70000
60000
50000
40000
30000
20000
10000
0
150
100
50
ppm (t1)

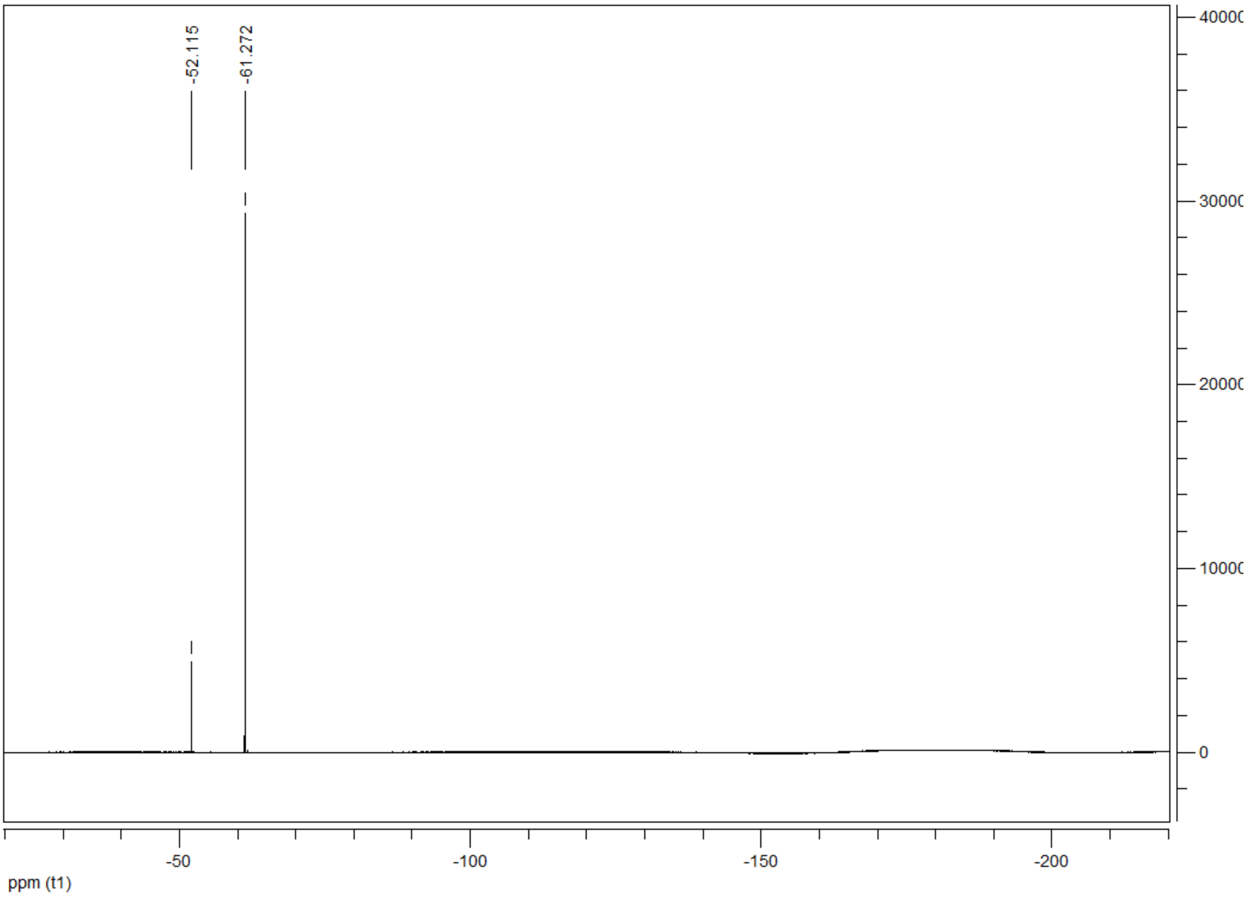
-52.115
-61.272
40000
30000
20000
10000
0
-50
-100
-150
-200
ppm (t1)

^{1}H, ^{13}C and ^{19}F NMR spectra of compound **9** (DMSO-d6),

4-((6-amino-9H-purin-9-yl)methyl)-N-(3-(trifluoromethyl)phenyl)benzamide:

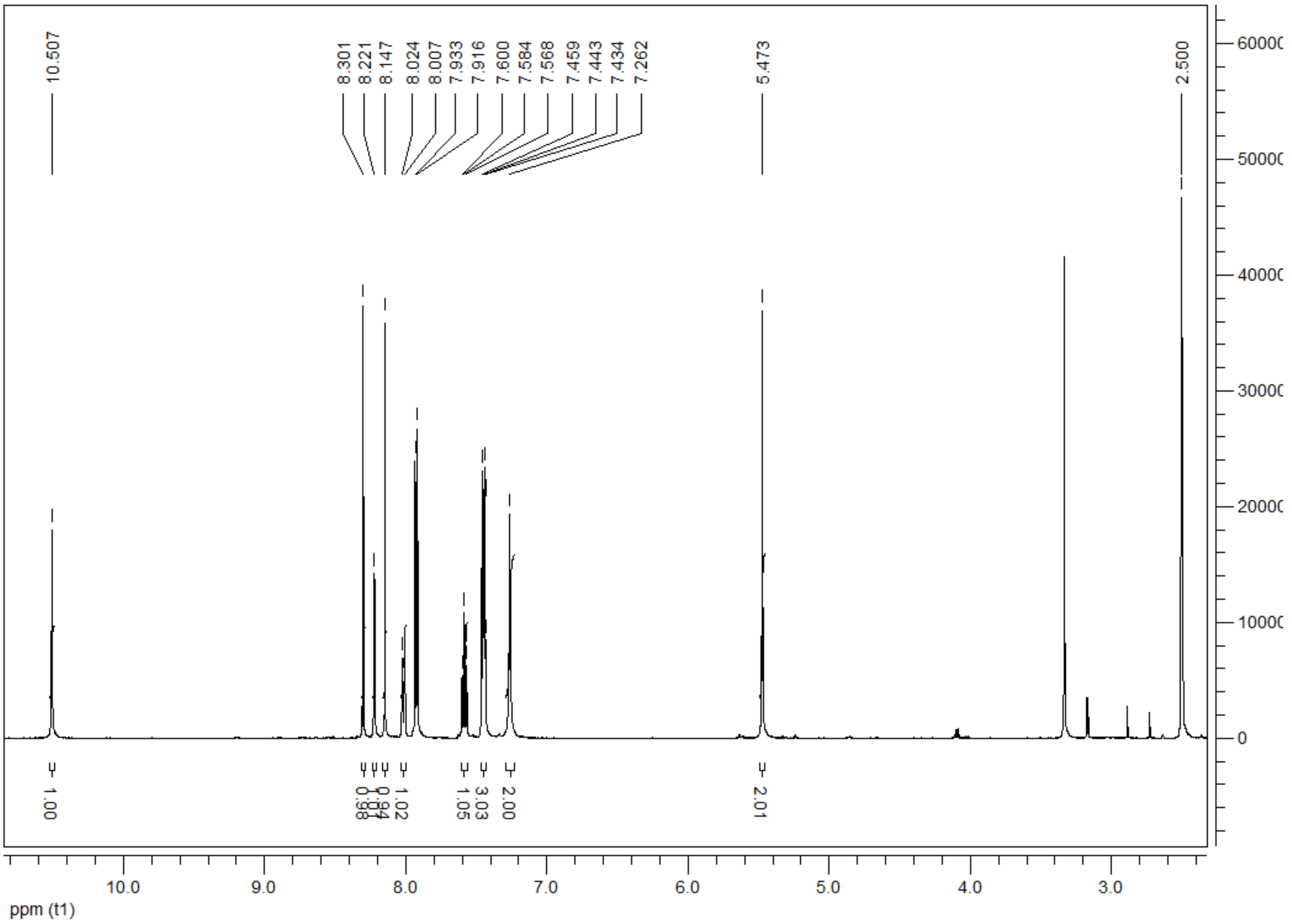

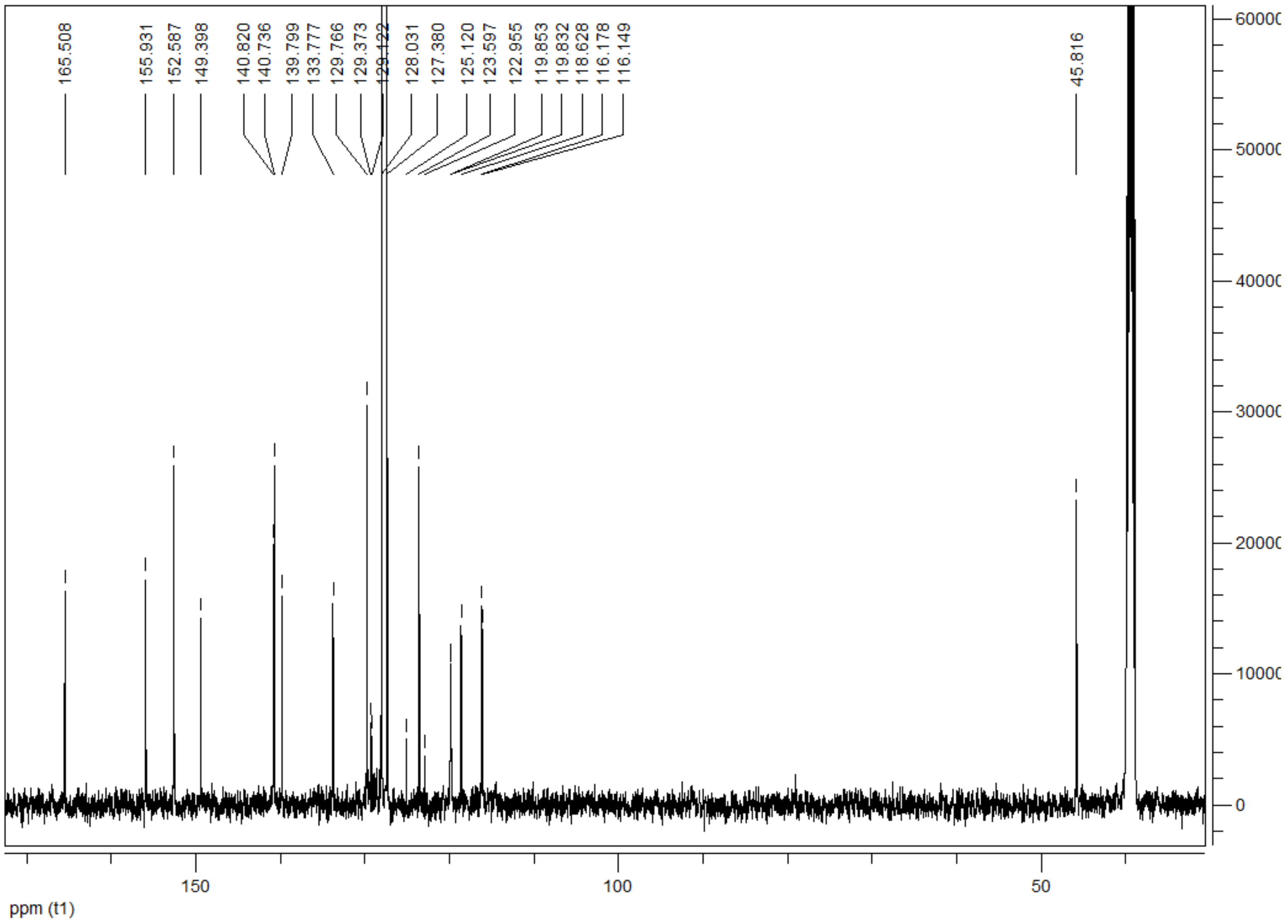

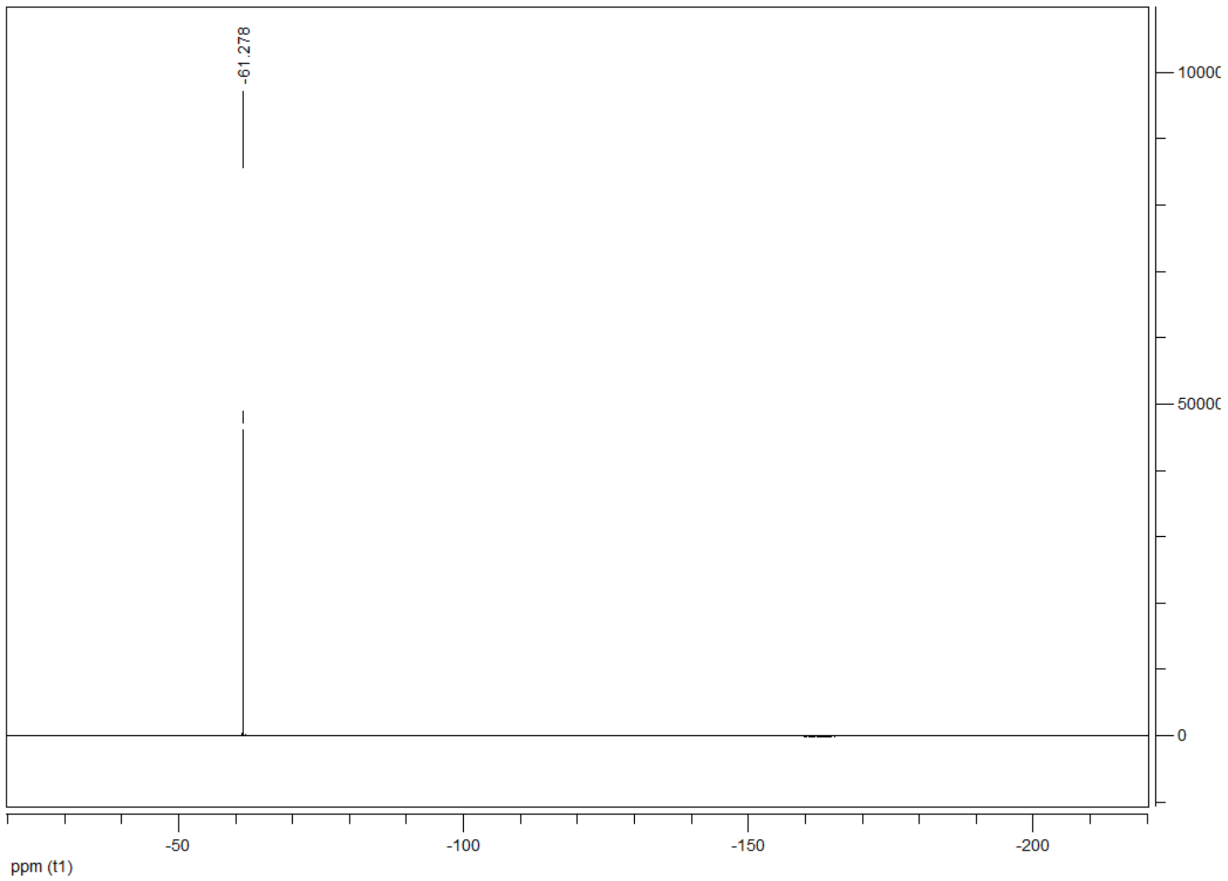

^{1}H, ^{13}C and ^{19}F NMR spectra of compound **10** (DMSO-d6),
4-((2,6-dichloro-9H-purin-9-yl)methyl)-N-(3-(4-methyl-1H-imidazol-1-yl)-5-(trifluoromethyl)-phenyl)benzamide:

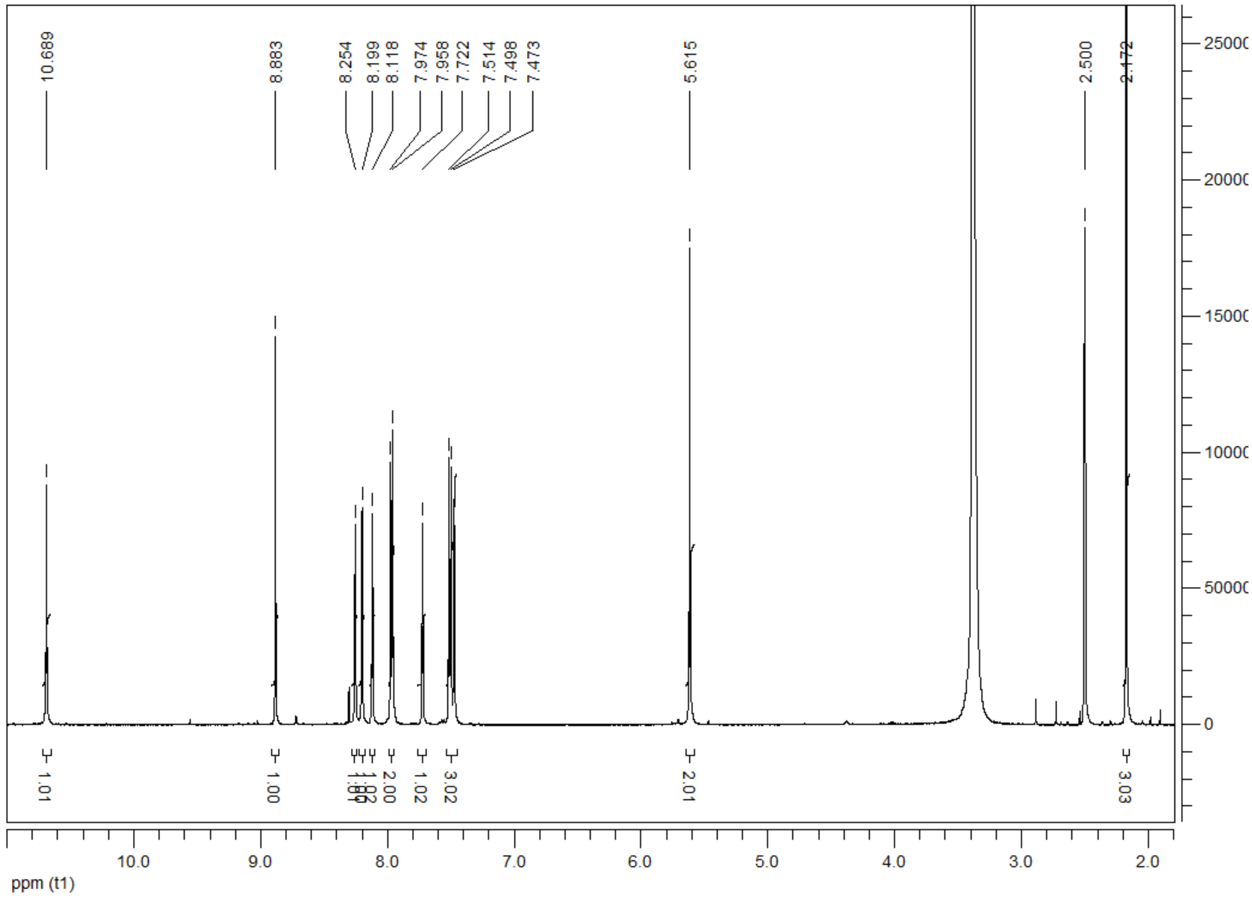

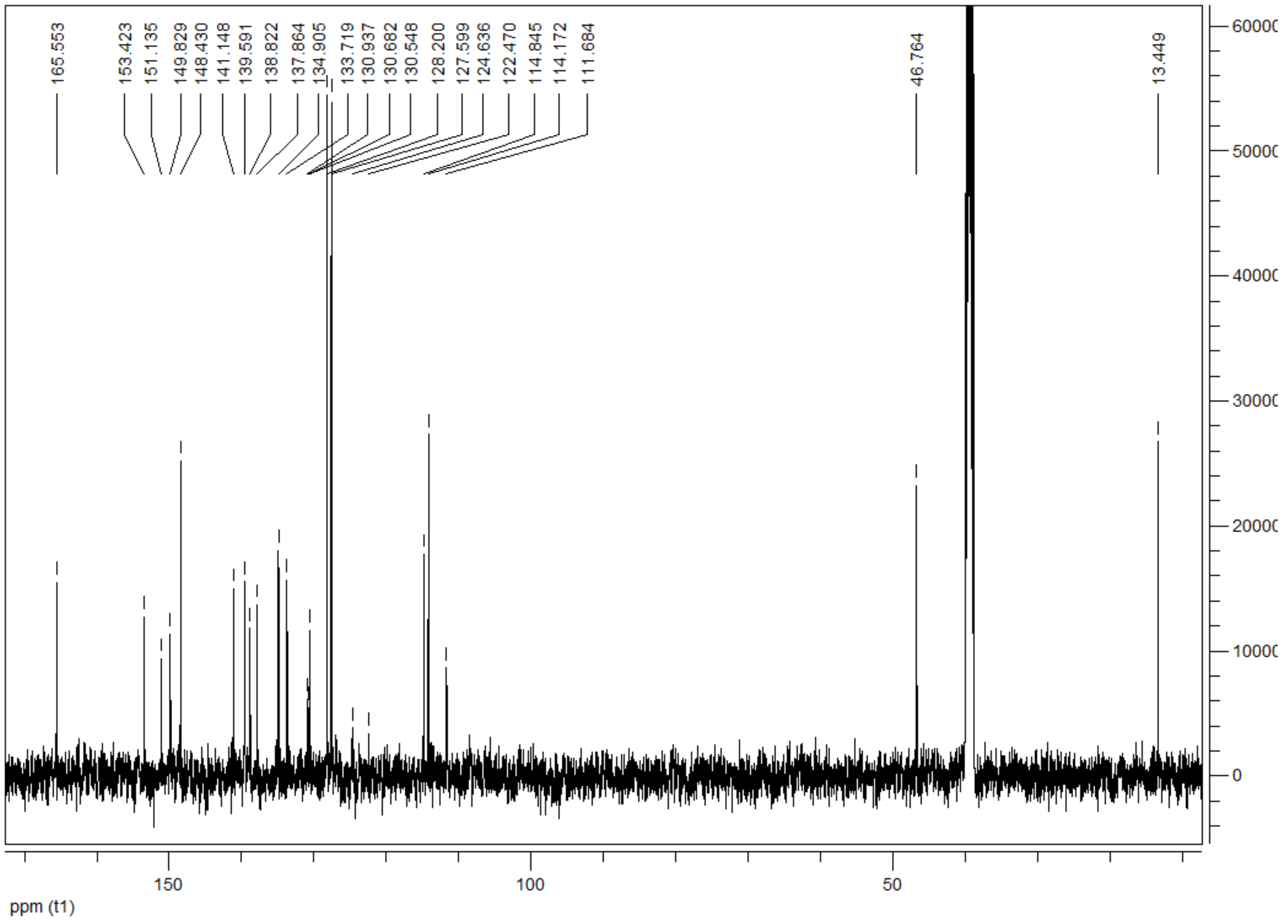
165.553
153.423
151.135
149.829
148.430
141.148
139.591
138.822
137.864
134.905
133.719
130.937
130.682
130.548
128.200
127.599
124.636
122.470
114.845
114.172
111.684
46.764
13.449
150
100
50
ppm (t1)

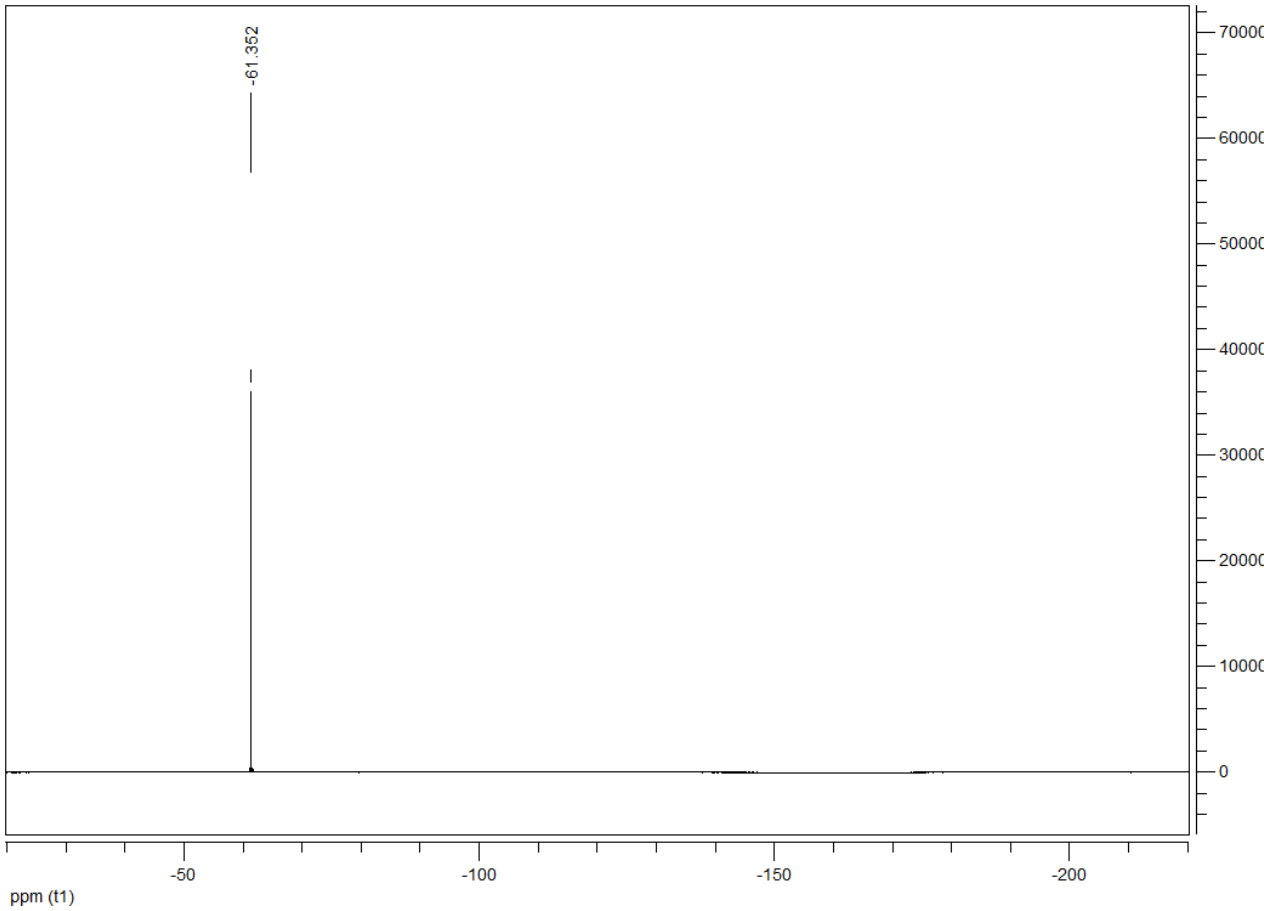
-61.352
-50
-100
-150
-200
ppm (t1)

^{1}H, ^{13}C and ^{19}F NMR spectra of compound **11** (DMSO-d6),

4-((6-amino-2-fluoro-9H-purin-9-yl)methyl)-N-(3-(4-methyl-1H-imidazol-1-yl)-5-(trifluoromethyl)phenyl)benzamide:

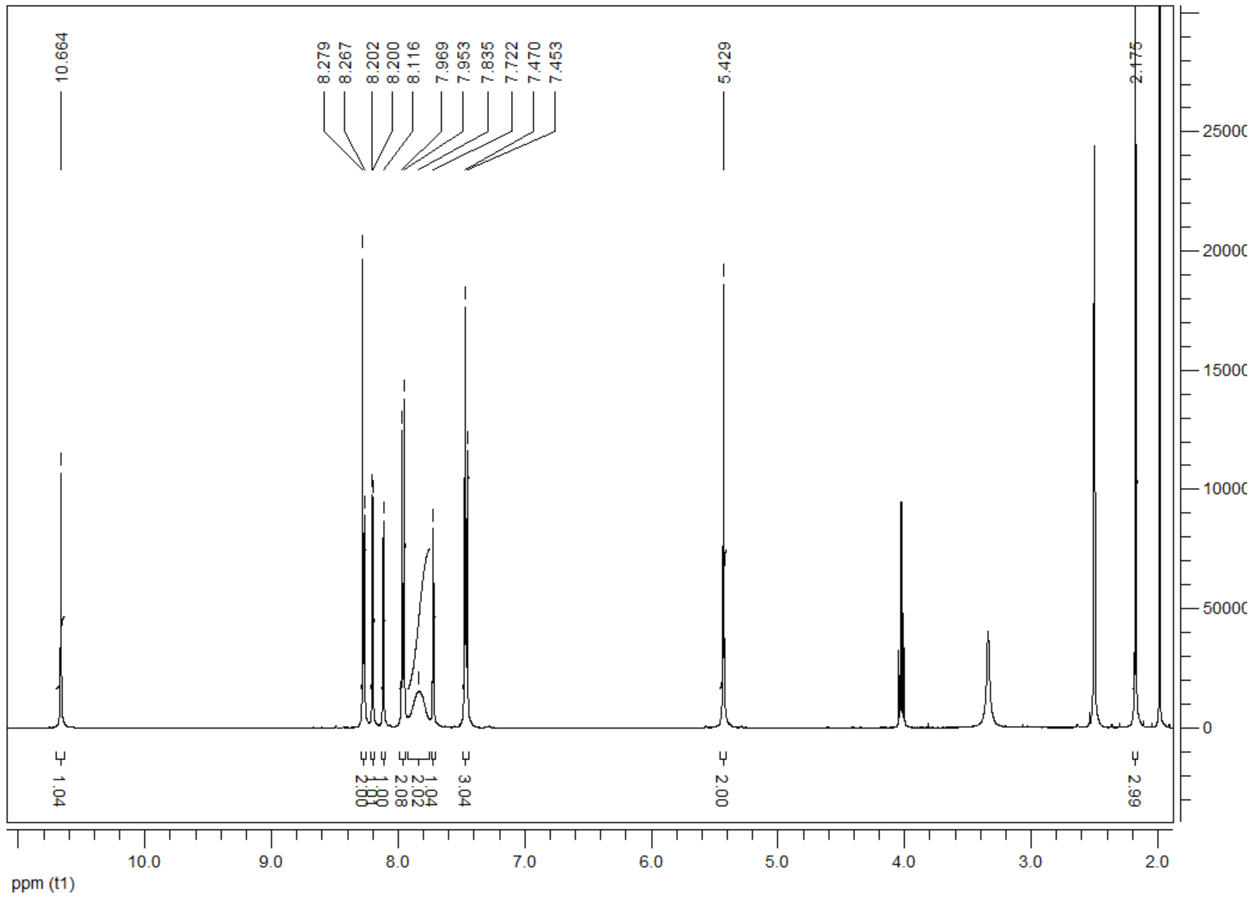

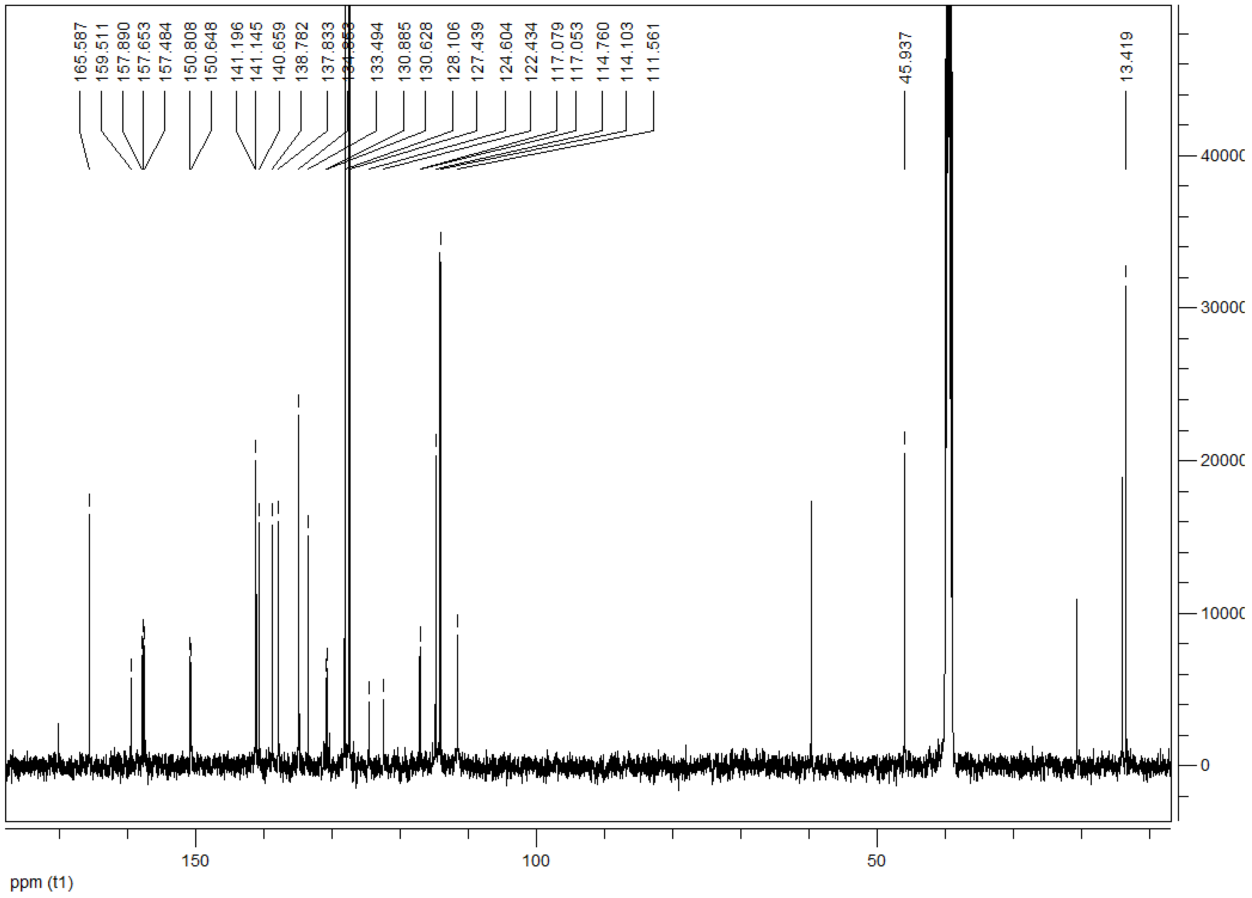

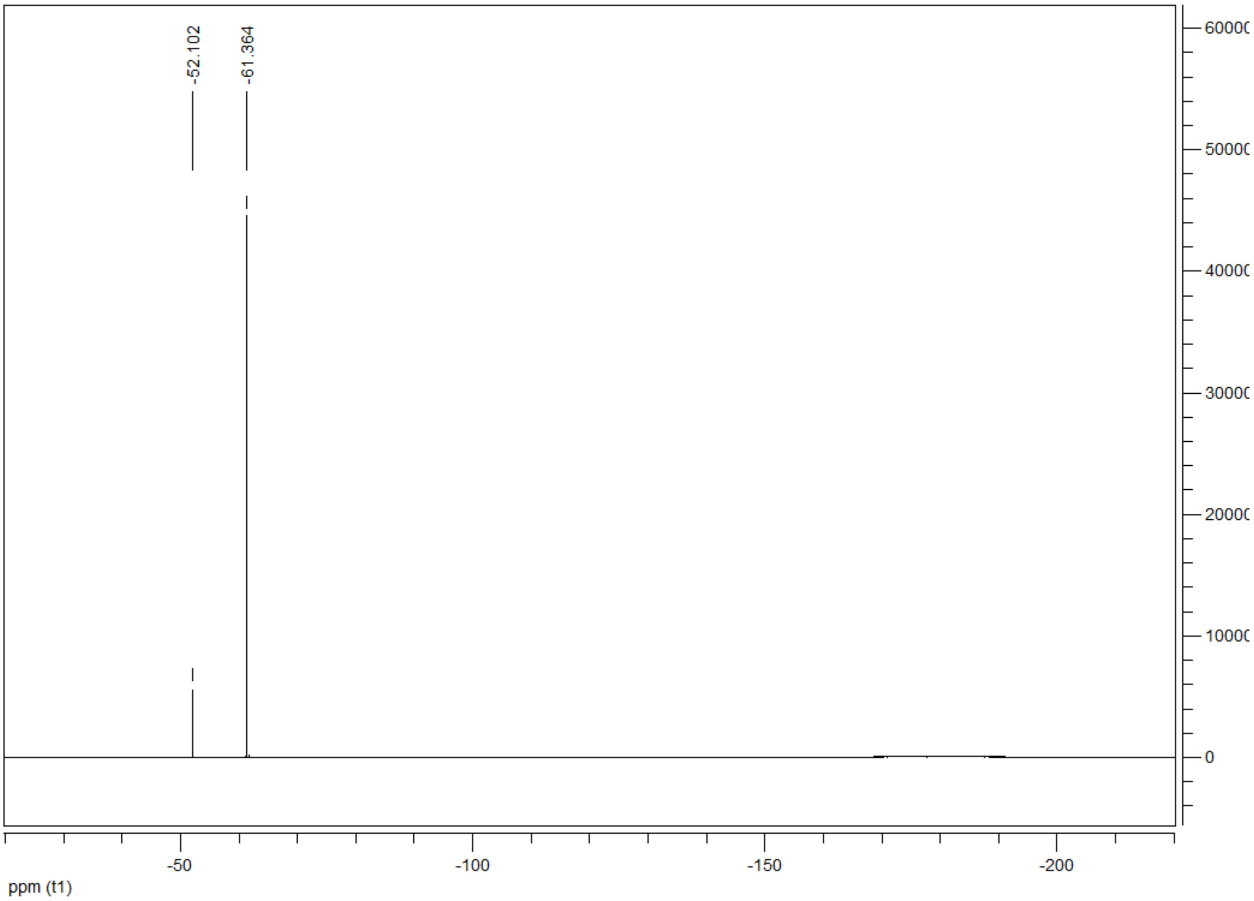

^{1}H, ^{13}C and ^{19}F NMR spectra of compound **12** (DMSO-d6),

4-((6-amino-9H-purin-9-yl)methyl)-N-(3-(4-methyl-1H-imidazol-1-yl)-5-(trifluoromethyl)phenyl)benzamide:

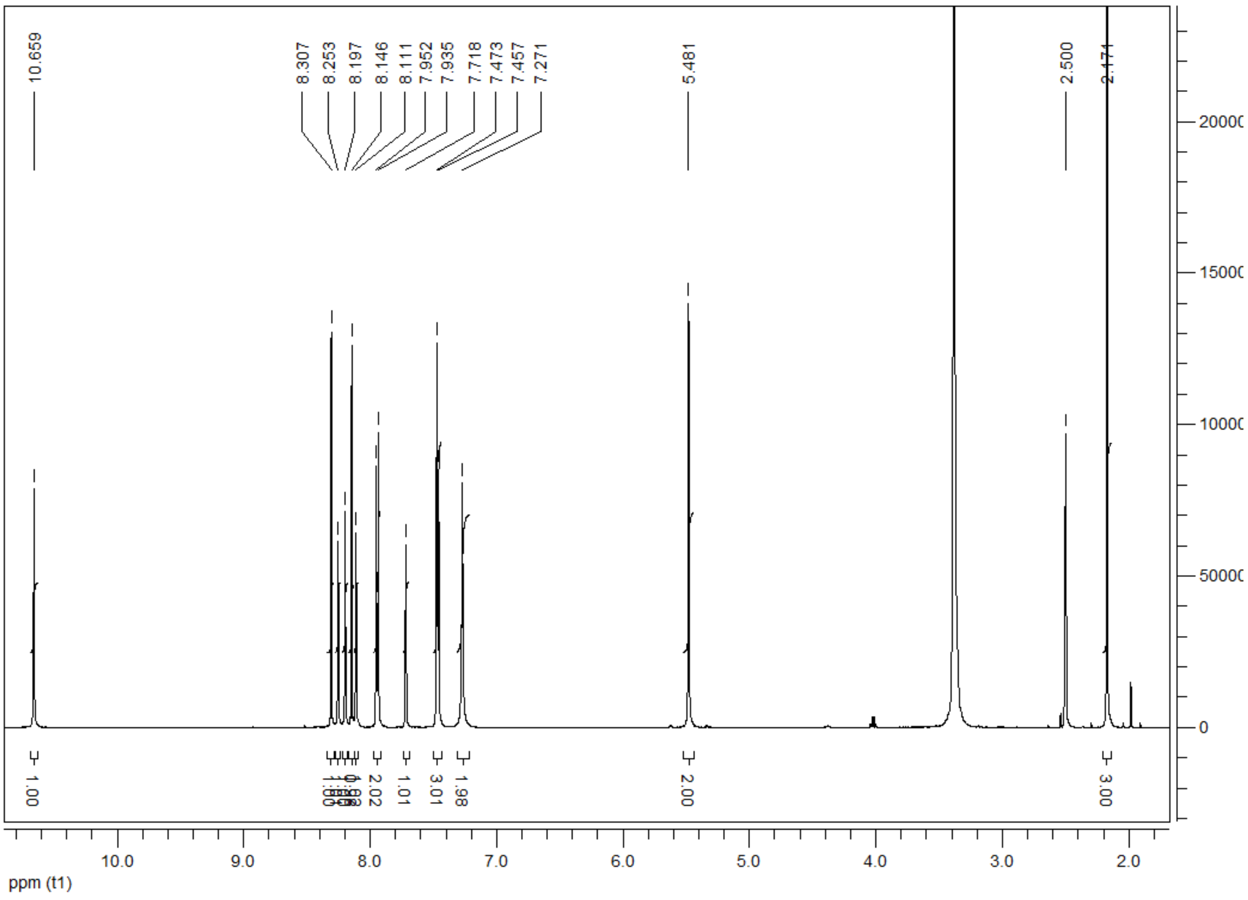

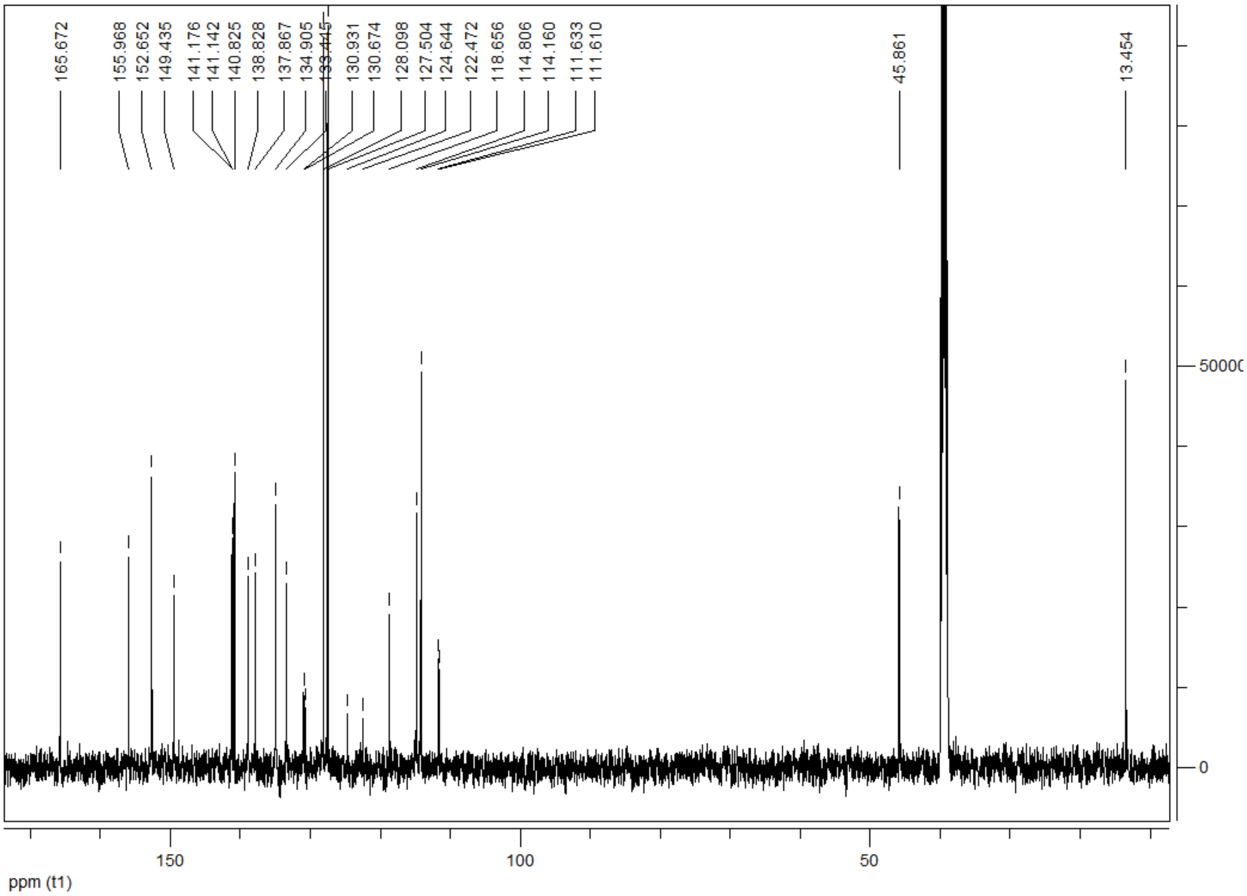
165.672
155.968
152.652
149.435
141.176
141.142
140.825
138.828
137.867
134.905
133.415
130.931
130.674
128.098
127.504
124.644
122.472
118.656
114.806
114.160
111.633
111.610
45.861
13.454
50000
0
150
100
50
ppm (t1)

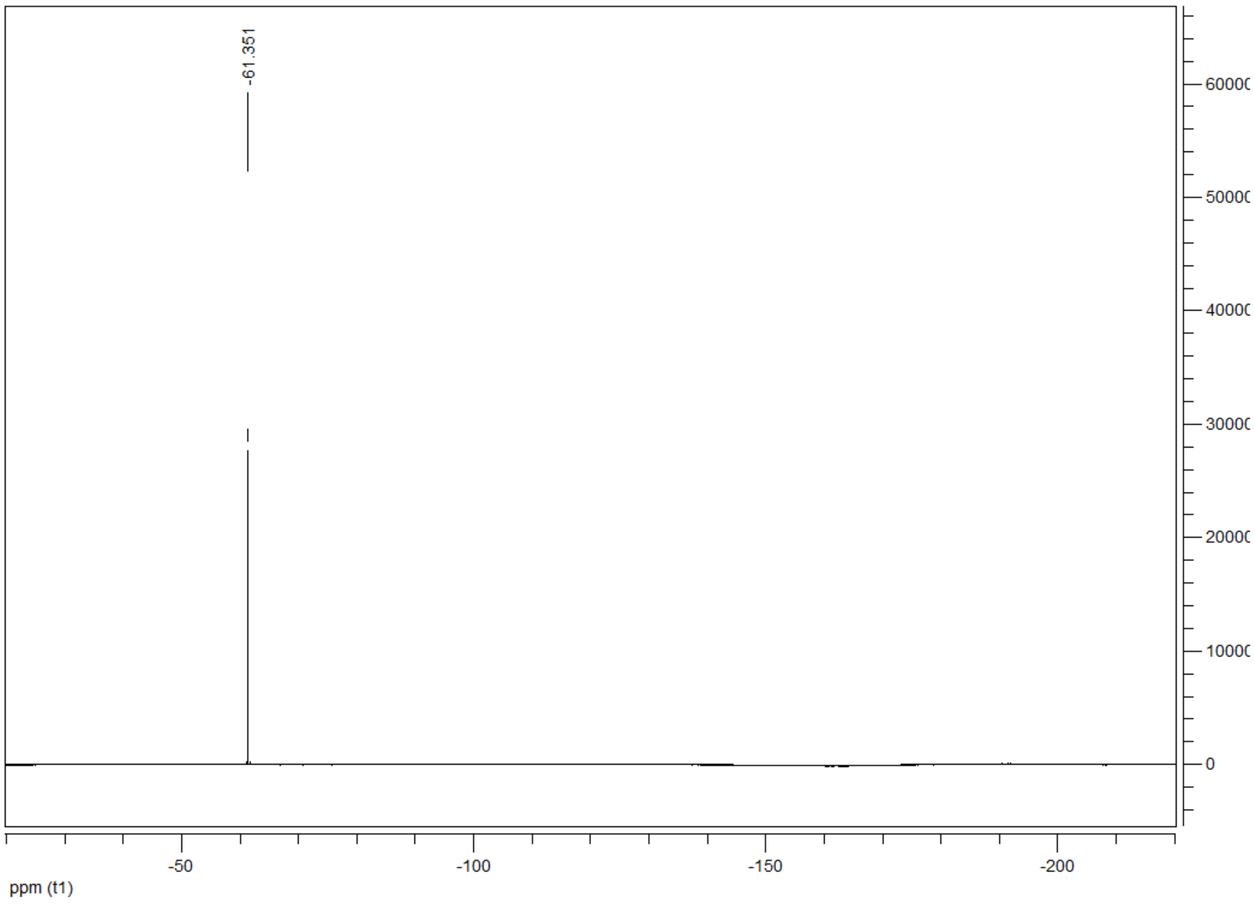
-61.351
60000
50000
40000
30000
20000
10000
0
-50
-100
-150
-200
ppm (t1)

^{1}H, ^{13}C and ^{19}F NMR spectra of compound **13** (DMSO-d6),

4-((2-chloro-6-methoxy-9H-purin-9-yl)methyl)-N-(3-(trifluoromethyl)phenyl)benzamide:

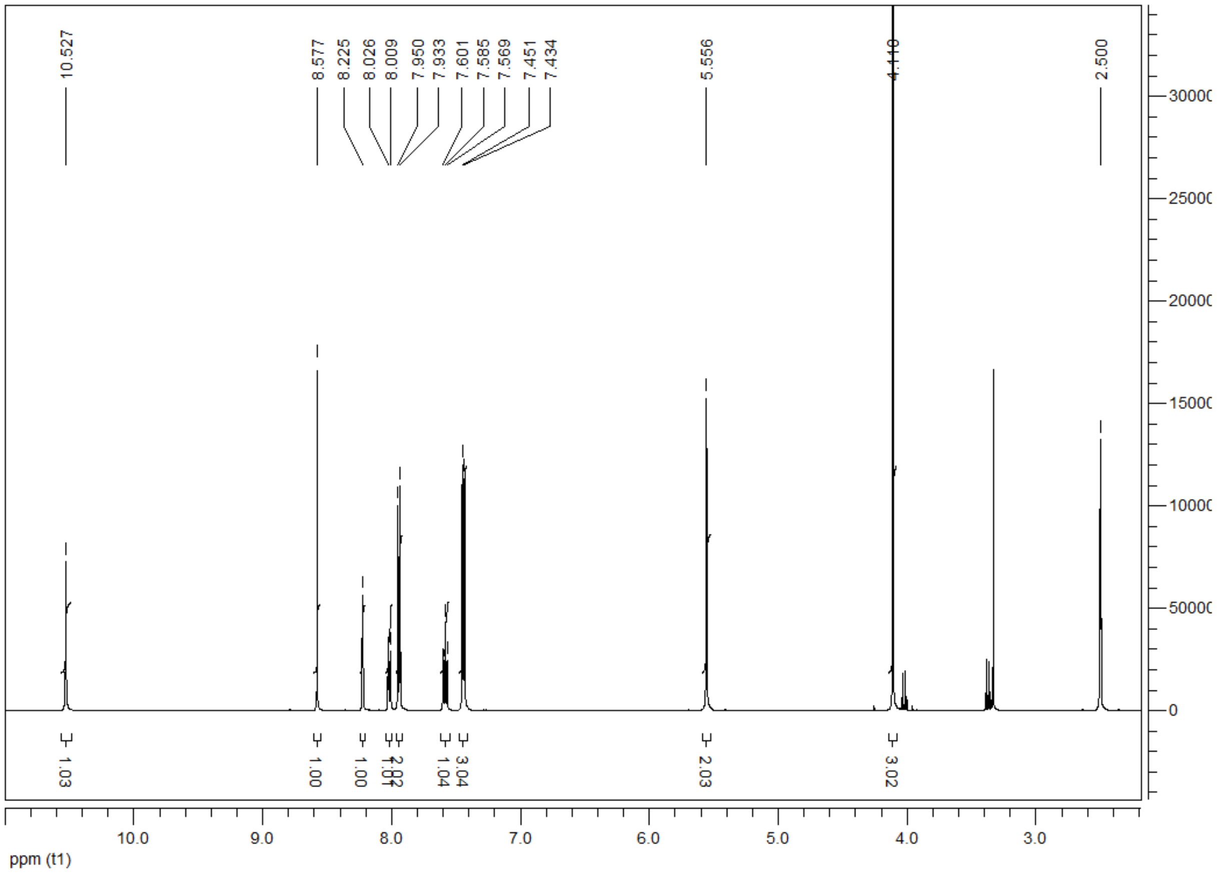

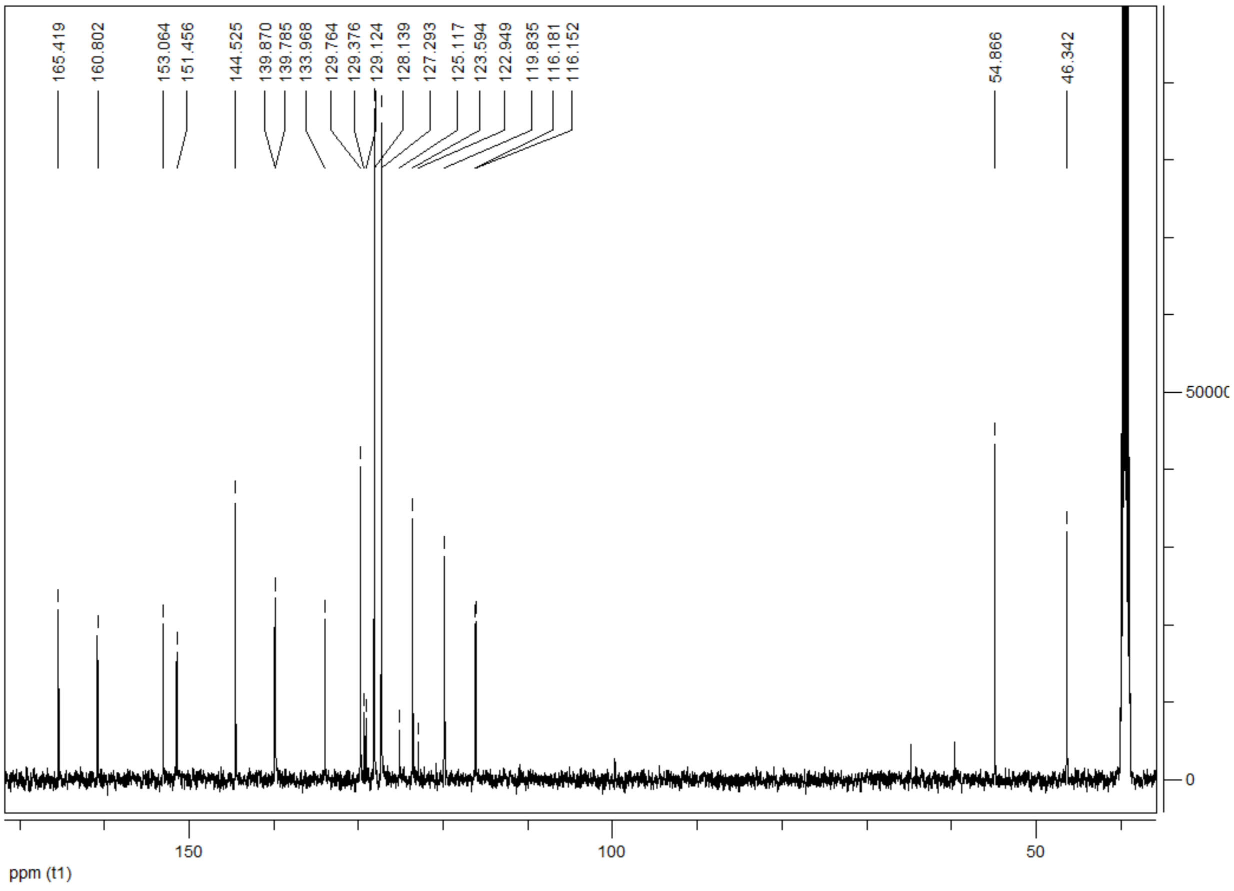

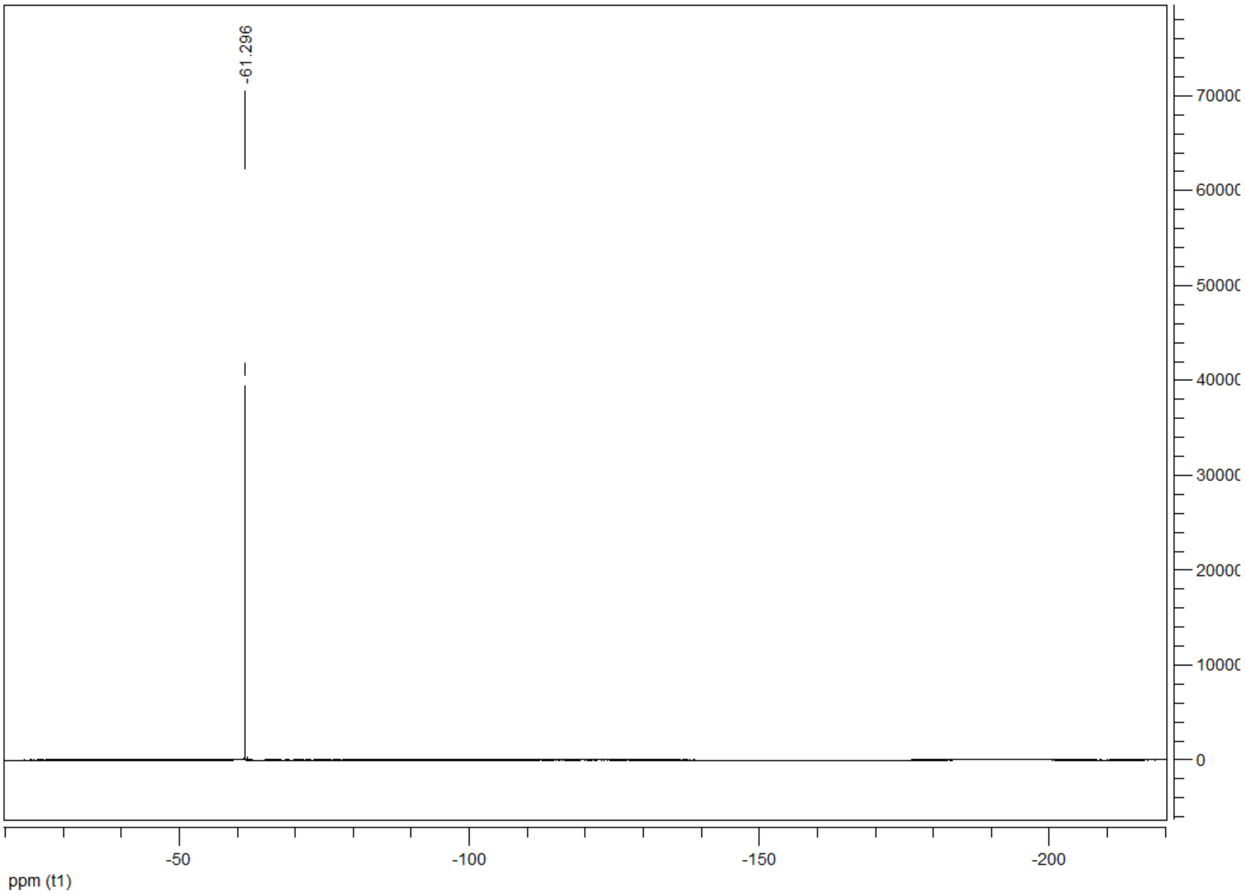

^{1}H, ^{13}C and ^{19}F NMR spectra of compound **14** (DMSO-d6),

4-((2-chloro-6-methoxy-9H-purin-9-yl)methyl)-N-(3-(4-methyl-1H-imidazol-1-yl)-5-(trifluoromethyl)phenyl)benzamide:

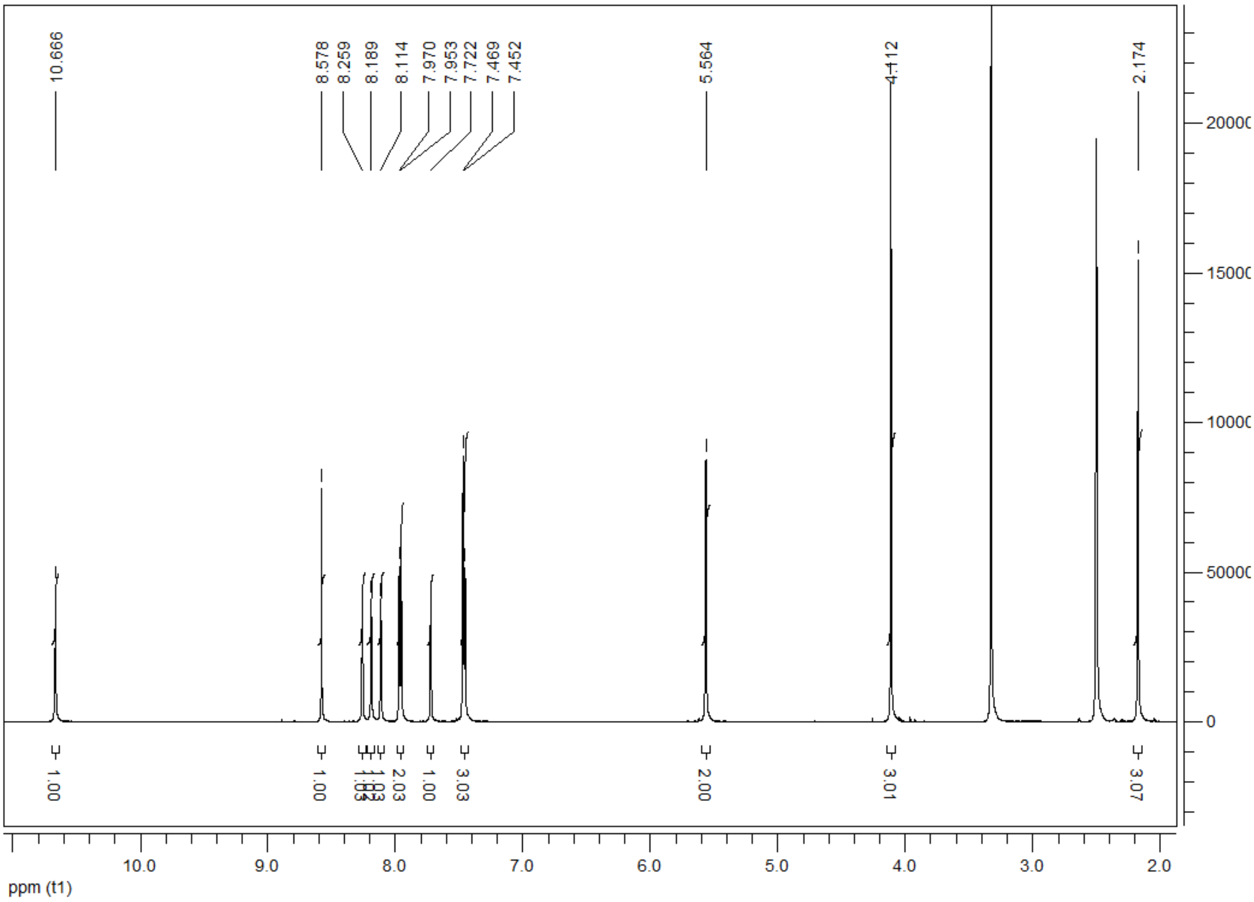

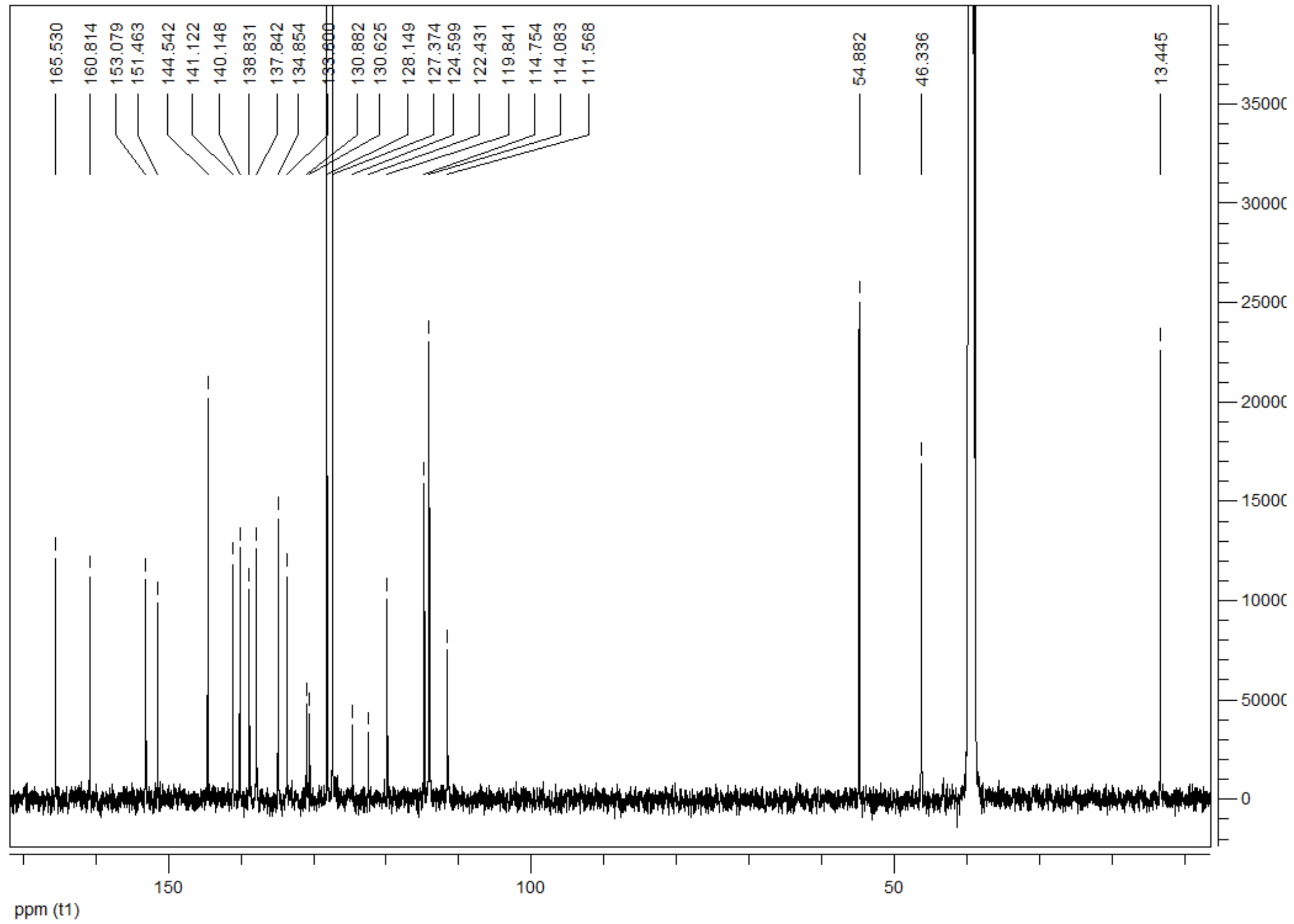
165.530
160.814
153.079
151.463
144.542
141.122
140.148
138.831
137.842
134.854
133.600
130.882
130.625
128.149
127.374
124.599
122.431
119.841
114.754
114.083
111.568
54.882
46.336
13.445
ppm (t1)

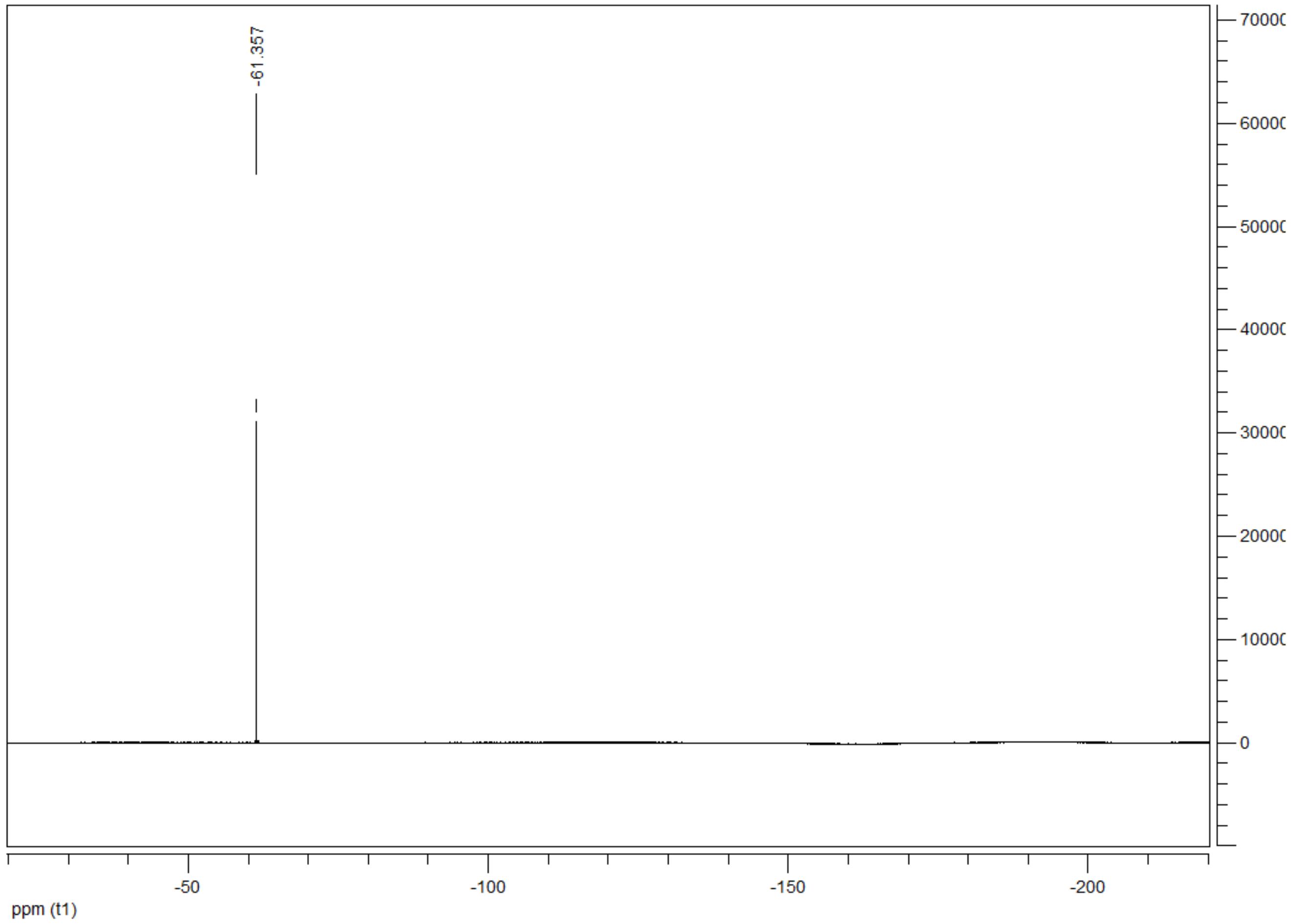
-61.357
ppm (t1)

^{1}H, ^{13}C and ^{19}F NMR spectra of compound **15** (DMSO-d6),

4-((6-amino-2-chloro-9H-purin-9-yl)methyl)-N-(3-(trifluoromethyl)phenyl)benzamide:

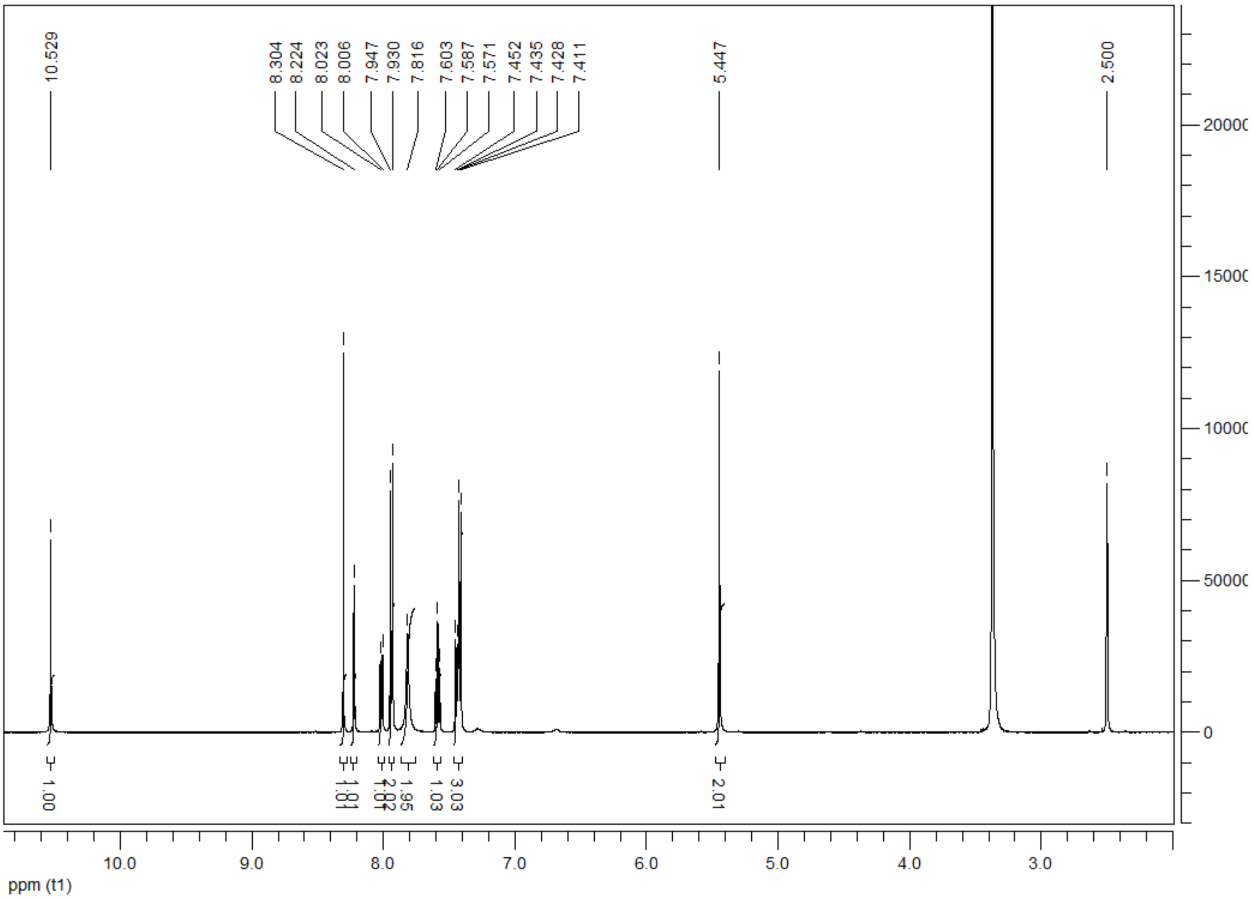

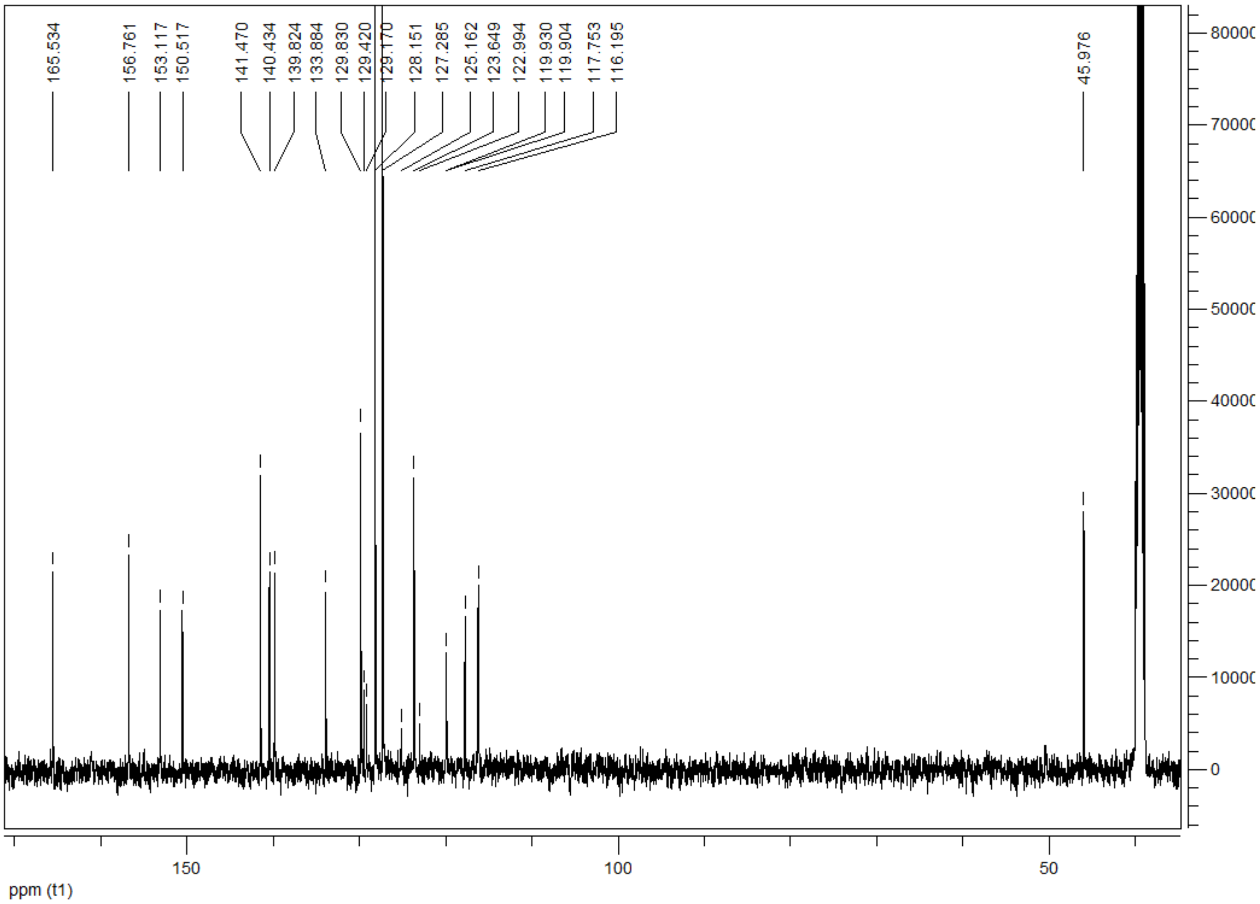

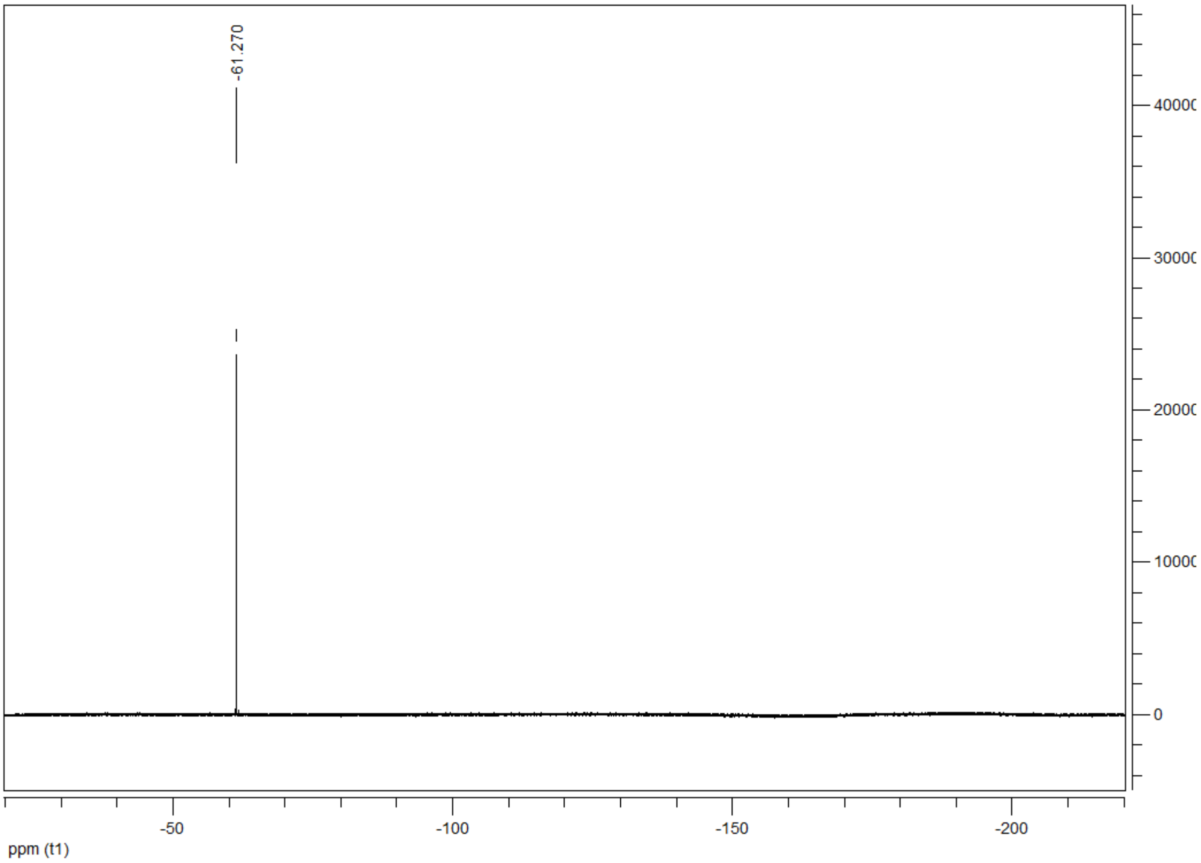

^{1}H, ^{13}C and ^{19}F NMR spectra of compound **16** (DMSO-d6),

4-((6-amino-2-chloro-9H-purin-9-yl)methyl)-N-(3-(4-methyl-1H-imidazol-1-yl)-5-(trifluoromethyl)phenyl)benzamide:

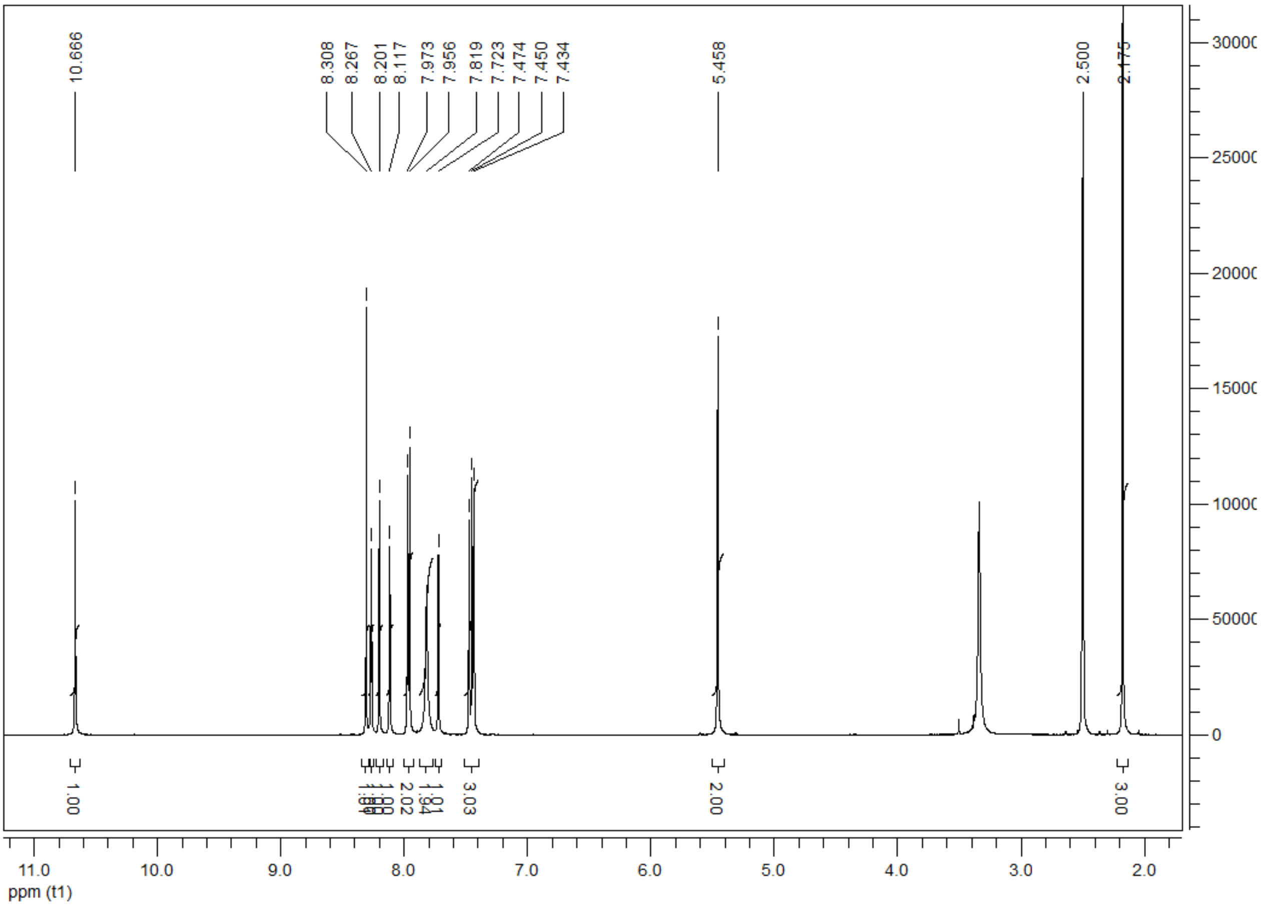

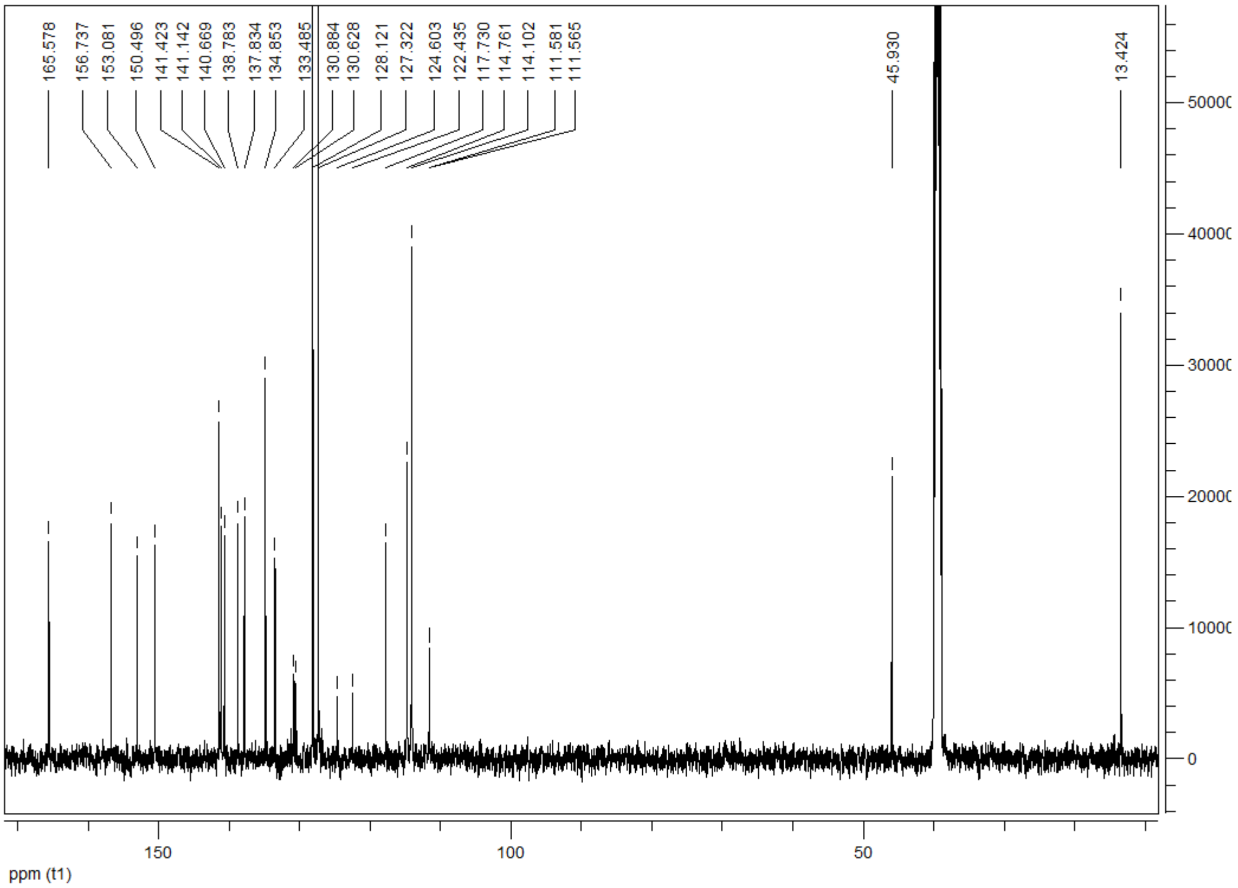
165.578
156.737
153.081
150.496
141.423
141.142
140.669
138.783
137.834
134.853
133.485
130.884
130.628
128.121
127.322
124.603
122.435
117.730
114.761
114.102
111.581
111.565
45.930
13.424
50000
40000
30000
20000
10000
0
150
100
50
ppm (t1)

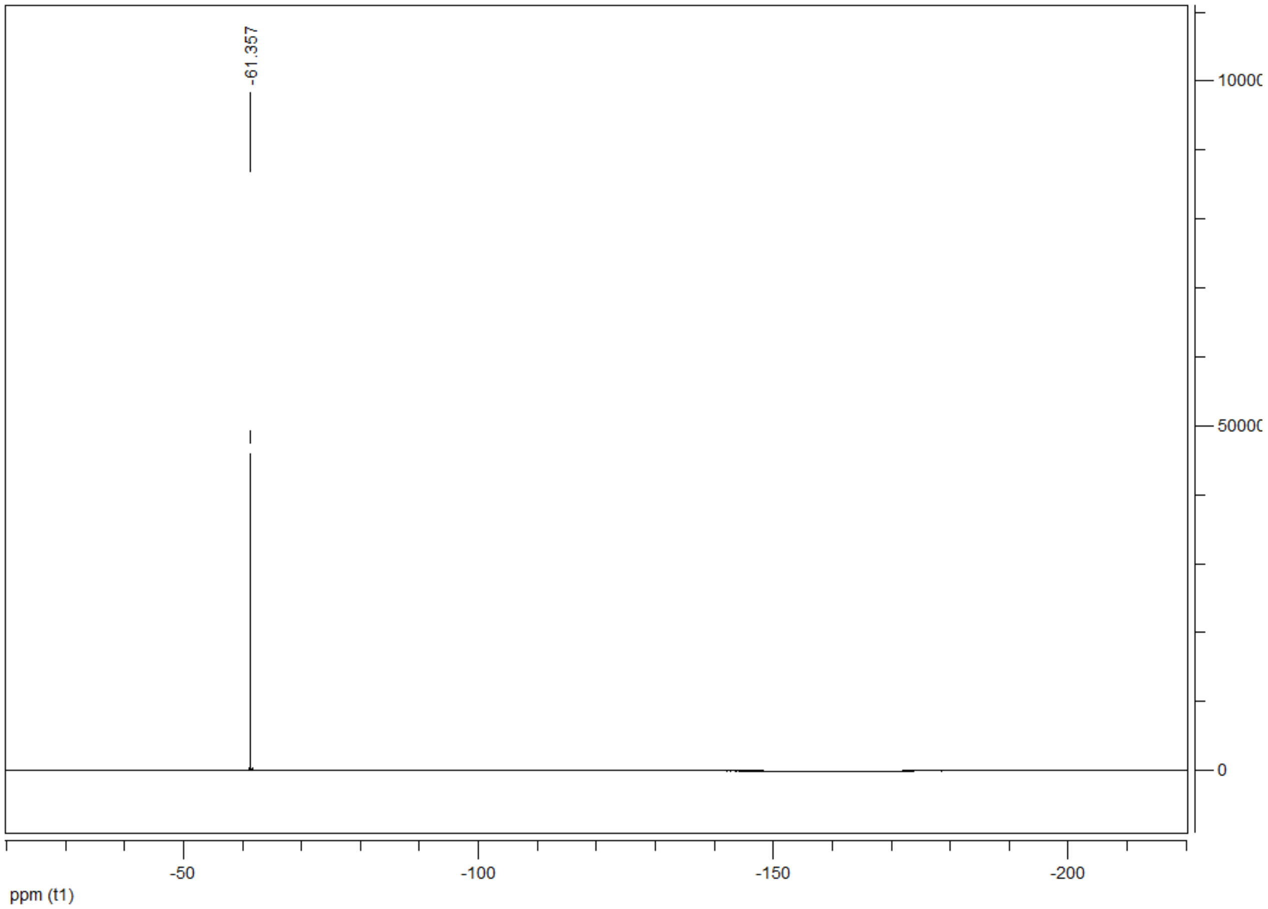
-61.357
10000
50000
0
-50
-100
-150
-200
ppm (t1)
